# Ret finger protein deficiency attenuates adipogenesis in male mice with high fat diet-induced obesity

**DOI:** 10.1038/s12276-025-01553-7

**Published:** 2025-09-18

**Authors:** Yun-Gyeong Lee, Anna Jeong, Yongwoon Lim, Sera Shin, Hosouk Joung, Hye Jung Cho, Su-Jin Lee, Hwang Chan Yu, Hyung-Seok Kim, Kwang-Il Nam, Gwang Hyeon Eom, Byung-Hyun Park, So-Young Park, Duk-Hwa Kwon, Hyun Kook

**Affiliations:** 1 Department of Pharmacology, Chonnam National University Medical School, Hwasun, Republic of Korea; 2 Chonnam University Resaserch Institute of Medical Science, Chonnam National University Medical School, Hwasun, Republic of Korea; 3 BioMedical Sciences Graduate Program, Chonnam National University, Hwasun, Republic of Korea; 4 BK21 plus Center for Creative Biomedical Scientists, Chonnam National University, Gwangju, Republic of Korea; 5 Department of Anatomy, Chonnam National University Medical School, Hwasun, Republic of Korea; 6 Department of Forensic Medicine, Chonnam National University Medical School, Hwasun, Republic of Korea; 7 Graduate School of Medical Science and Engineering, Korea Advanced Institute of Science and Technology, Daejeon, Republic of Korea; 8 Department of Physiology, College of Medicine, Yeungnam University, Daegu, Republic of Korea

**Keywords:** Transcriptional regulatory elements, Obesity, Disease model

## Abstract

Ret finger protein (RFP, also known as TRIM27) is a multifunctional E3 ubiquitin ligase implicated in transcriptional regulation. While previously reported to repress myogenesis, its role in adipose tissue metabolism remains unclear. Here, using both global and adipocyte-specific RFP-knockout male mice subjected to high-fat diet feeding, we found that RFP deficiency markedly attenuated body weight gain, adipose tissue expansion and adipocyte hypertrophy, while improving glucose tolerance, insulin sensitivity and circulating lipid profiles. Indirect calorimetry demonstrated significantly increased whole-body energy expenditure, independent of food intake or physical activity. Consistent with these murine findings, RFP expression was elevated in omental adipose tissue from obese human subjects, underscoring the translational relevance. Mechanistic studies revealed that RFP physically interacts with PPAR-γ and enhances its transcriptional activity, thereby promoting expression of adipogenic target genes such as AP2 and adiponectin. Loss of RFP suppressed adipocyte differentiation both in vivo and in vitro. Collectively, our findings identify RFP as a positive regulator of adipogenesis and systemic metabolism via PPAR-γ activation. Genetic ablation of RFP confers resistance to high-fat diet-induced obesity, suggesting that targeting RFP may represent a potential therapeutic strategy for obesity and related metabolic disorders.

## RFP deficiency reduces obesity and enhances metabolism

Obesity is a growing global issue, leading to health problems such as diabetes and heart disease. Researchers are exploring new ways to tackle obesity by studying the role of a protein called Ret finger protein (RFP). RFP is known for its involvement in various cellular processes, including muscle development and protein regulation. This study investigates the role of RFP in fat cell development and its potential as a target for obesity treatment. The researchers used mice genetically modified to lack RFP and fed them a high-fat diet to study obesity. They found that mice without RFP gained less weight and had better glucose metabolism compared with normal mice. This suggests that RFP plays a role in fat accumulation and metabolism. The study also showed that RFP interacts with PPAR-γ, a key regulator of fat cell development, enhancing its activity.

This summary was initially drafted using artificial intelligence, then revised and fact-checked by the author.

## Introduction

The global burden of obesity and its related metabolic disorders has reached epidemic proportions, with the World Health Organization reporting that 43% of adults worldwide are overweight and 16% are obese^1,2^. These conditions important exaggerate the risk of various complications, such as type 2 diabetes, cardiovascular diseases and certain types of cancers^3^. Adipose tissue, previously considered merely a passive energy storage organ, is now recognized as a dynamic endocrine organ that plays an important role in the maintenance of whole-body energy homeostasis^4^. Thus, the complex process of adipogenesis, a process of differentiation of preadipocytes into mature adipocytes, contributes the appropriate functions of adipose tissue.

Ret finger protein (RFP), first identified as a gene involved in the RET oncogene rearrangement, is a member of the tripartite motif (TRIM, thus also known as TRIM27) protein family characterized by its typical structure of RING finger, B-box and coiled-coil domains^5,6^. RFP has been implicated in diverse cellular processes including innate immunity^7^, cell proliferation^8^ and transcriptional regulation^9^. RFP has E3 ubiquitin ligase activity with its RING finger domain, which enables the regulation of protein stability by ubiquitination-mediated proteasomal degradation^6,10^. Additionally, RFP functions as a transcriptional regulator through its interactions with diverse transcription factors^11^.

Our previous works revealed a crucial role for RFP in skeletal muscle differentiation, where it acts as a negative regulator of myogenesis through dual mechanism: acting as a transcriptional repressor^12^ and functioning as an E3 ligase to promote Pax7-mediated MyoD degradation^13^. Interestingly, in our investigation on the role of RFP in muscle injury, we observed a notable increase in intramuscular vacuoles^13^ in RFP knockout (KO) mice after muscle injury, which were suspected to contain lipids. These results suggest the potential involvement of RFP in lipid metabolism, which led us to hypothesize that RFP might serve as a regulator coordinating the balance between myogenic and adipogenic differentiation. However, despite these implications, the specific role of RFP in lipid metabolism and adipose tissue regulation remains largely unexplored. Given the need for novel therapeutic targets in obesity and metabolic disorders, our preliminary observations of potential role of RFP in lipid metabolism require further investigation. Here, we demonstrate that RFP functions as a key regulator of adipogenesis through its interaction with PPAR-γ and that genetic ablation of RFP protects against diet-induced obesity and metabolic dysfunction. Our findings identify RFP as a promising therapeutic target for obesity and related metabolic disorders, providing new insights into the molecular mechanisms in adipose tissue regulation.

## Materials and methods

### Animals

All animal experiments were approved by the Institutional Animal Care and Use Committee of Chonnam National University Medical School (nos. CNU-IACUC-H-2023-42 and CNU-IACUC-H-2024-4) and followed the Guide for the Care and Use of Laboratory Animals (eighth edition, National Academies Press). RFP KO mice were as previously described^13^. Adult male RFP KO mice and wild-type (WT) littermates (8−10 weeks old) were randomly divided into two groups, and provided with either a normal diet (ND; RodFeed) or a 60% high-fat diet (HFD; D12492, Research Diets) during 10 or 12 weeks. Food intake and body weight were monitored once every 3 days throughout the study.

*RFP*^*flox/flox*^ mice (B6.*Trim27*^*em1flox*^/N) were generated by Cyagen Biosciences and *Adipoq-Cre* mice were obtained from Dr. Hail Kim (Korea Advanced Institute of Science and Technology). To create adipocyte-specific RFP conditioned KO mice (cKO; *Adipoq-Cre;RFP*^*flox/flox*^), we performed crosses between *RFP*^*flox/flox*^ and *Adipoq-Cre* mice. HFD feeding was carried out in the same manner as with RFP KO male mice.

Intraperitoneal glucose tolerance tests (2.5 g/kg of body weight) and insulin tolerance tests (0.75 U/kg of body weight) were performed on male mice after a 6 h fast as described previously^14^. Tail vein blood glucose levels were measured at 0, 15, 30, 60, 90 and 120 min after injection using a handheld glucometer (Bayer). The data were subsequently graphed as a baseline-corrected area of the curve (AOC), as previously described^15^.

Indirect calorimetry was performed on male mice after HFD using an Oxymax/CLAMS metabolic cage system (Columbus Instruments). Each mouse was housed individually in a chamber, as described previously^16^. Following a 24-h acclimatization period to the metabolic cages, key metabolic parameters including respiratory exchange ratio (VCO_2_/VO_2_), energy expenditure, food intake and physical activity were continuously monitored for 48 h. Experiments were conducted under ad libitum feeding conditions in an environmental room maintained at 20–23 °C with a 12-h light–dark cycle (6:00–18:00).

### Human samples

For the quantitative PCR with reverse transcription (RT−qPCR) analysis of adipogenic and lipogenic gene expression, omental fat tissues were isolated from postmortem human specimens at the Department of Forensic Medicine, Chonnam National University Medical School (IRB no. 906-250219-BR-013-02).

For the confirmation of RFP intracellular localization, omental fat specimens were obtained from human cadaveric donors during educational dissections conducted by medical students at the Department of Anatomy, Chonnam National University Medical School. The dissection procedures adhered to the ethical principles outlined in the Declaration of Helsinki (64th WMA General Assembly) and followed both local and international guidelines for anatomical research using human cadaveric donors^17^.

### Histology

Mouse fat tissues were fixed in a 4% paraformaldehyde. Subsequently, paraffin blocks were cut into sections with a thickness of 8 μm. Fat tissue sections were stained with H&E for examination under a light microscope. To assess adipocyte area within the chosen fat tissue sections, ZEN blue software (Carl Zeiss) was used. For immunofluorescence staining, sections were deparaffinized and then immunostained with antibodies against Plin1 (Progen) or RFP (IBL). After washing with PBS, secondary antibodies (Alexa Fluor 488-conjugated goat anti-mouse or Alexa Fluor 568-conjugated goat anti-rabbit, Invitrogen) were incubated overnight at 4 °C. Sections were then counterstained with 4′,6-diamidino-2-phenylindole (DAPI; Invitrogen).

### Biochemical analysis

Male mice were humanely euthanized using carbon dioxide after overnight fasting for biochemical analysis, in accordance with the AVMA Guidelines for the Euthanasia of Animals (2020 edition). Plasma levels of various analytes were determined using specific kits as per the manufacturer’s instructions. The following parameters were measured: insulin (Invitrogen), free fatty acid (FFA; Abcam), glycerol (Sigma-Aldrich), leptin (Invitrogen), adiponectin (Invitrogen), triglyceride (TG; Cayman Chemical).

### mRNA sequencing analysis

To explore how RFP deficiency affects gene expression, we subjected WT and RFP KO male mice to a 12-week HFD regimen and utilized epididymal white adipose tissue (eWAT) for mRNA sequencing analysis. Total RNA was isolated using Trizol reagent (Invitrogen), and RNA quality and quantity were assessed using a TapeStation 4000 System (Agilent Technologies) and Qubit 4 fluorometer (Thermo Fisher Scientific), respectively. Libraries were prepared using the CORALL RNA-Seq V2 Library Prep kit (Lexogen), with mRNA isolated using the Poly(A) RNA selection kit (Lexogen). Indexed libraries were enriched by PCR, and quality was confirmed using TapeStation HS D1000 screen tape. Sequencing was performed on a NovaSeq 6000 platform (Illumina) with paired-end 100 sequencing.

Raw data quality control was conducted using FastQC and trimming was performed with Fastp. Reads were mapped to the reference genome using STAR and read quantification was processed with Salmon. Normalization and differential expression analysis were performed using the TMM + CPM method in Python’s conorm package, and visualization was conducted using ExDEGA (Ebiogen). mRNA expression data have been deposited in the GEO database under accession code GSE283657.

### Plasmids and siRNA

The *pFlag-CMV2-human RFP* (Flag−RFP) construct was obtained from Dr. Takahashi (Nagoya University School of Medicine). The *pCS4-HA-PPAR-γ* (HA−*PPAR-γ*) construct was obtained from Dr. Kwang Youl Lee (Chonnam National University School of Medicine)^18^. The siRNA against RFP was purchased from Bioneer. Cells were transfected with siRNA (60 nM) using Lipofectamine RNAi MAX (Invitrogen) according to the manufacturer’s instructions. The siRNA sequences are provided in Supplementary Table 1.

### Cell culture

The 3T3-L1 murine preadipocytes (CL-173) and HEK293T fibroblasts (CRL-3216) used for coimmunoprecipitation were obtained from the American Type Culture Collection (ATCC). Culture media were supplemented with antibiotics (penicillin and streptomycin, Life Technologies Corporation). Cells were cultured in DMEM containing 10% bovine calf serum (BCS) or fetal bovine serum (FBS). Cells were maintained at 37 °C in a humidified environment with 5% CO_2_. Primary adipocytes and stromal vascular fraction cells (SVCs) were isolated from eWATs of male mice through digestion with collagenase (type 2, Worthington Biochemical Corporation) as described previously^19^.

### Adipocyte differentiation

The 3T3-L1 preadipocytes and SVCs differentiation was induced as described previously^20^. The cells were allowed to grow for 2 days after reaching confluence in a culture medium consisting of DMEM−10% BCS. To induce the differentiation process, we introduced the differentiation medium, which included 0.5 mM 3-isobutyl-methylxanthine, 1 μM dexamethasone and 10 μg/ml insulin, into a DMEM−10% FBS culture medium. After an additional 2 days, the cells were maintained in DMEM−10% FBS with 10 μg/ml insulin for another 2 days. Following this, the culture medium was changed to DMEM−10% FBS every 2 days over the course of the next 4 days.

### Western blotting and coimmunoprecipitation

Western blotting and coimmunoprecipitation were performed as described previously^21^. Briefly, tissue homogenates and cell lysates were separated by SDS−PAGE using a 10−15% gel and transferred onto PVDF membranes overnight at 100 mA. Following blocking with 5% skim milk, the blots were incubated with primary antibodies: anti-RFP (1:500, IBL), anti-PPAR-γ (1:1,000, Cell Signaling Technology), anti-FABP4 (AP2, 1:1,000, Cell Signaling Technology), anti-adiponectin (1:1,000, Cell Signaling Technology), anti-Flag (1:5,000, Sigma-Aldrich), anti-HA (1:2,000, Sigma-Aldrich), anti-HSP90 (1:1,000, Cell Signaling Technology) and anti-β-actin (1:500, Sigma-Aldrich) overnight at 4 °C. After incubation with primary antibodies, the membranes were incubated in HRP-conjugated secondary anti-rabbit or anti-mouse antibodies for 1 h at 25 °C. Protein bands were detected using an Azure c300 (Azure Biosystems) chemiluminescent western blot imaging system. For coimmunoprecipitation, proteins were incubated with primary antibodies overnight at 4 °C. The immune complexes were precipitated using protein A/G beads for 2 h at 4 °C. After washing, the immunoprecipitates were separated by SDS−PAGE and immunoreactive bands were detected using an Azure c300 (Azure Biosystems).

### RNA isolation and qPCR

Total RNA was isolated from the cells or tissues using TRIzol reagent (Molecular Research Center). cDNA was synthesized using ReverTra Ace cDNA synthesis kits (Toyobo) and analyzed with qPCR using QuantiTect SYBR Green-based PCR kits (Qiagen) on a Rotor-Gene Q real-time PCR cycler (Qiagen). The relative expression of genes were calculated on the cycle threshold values using the 2^−ΔΔCt^ method. All data were normalized to GAPDH. The primers used for PCR are provided in Supplementary Table 1.

### Oil red O staining

Oil red O staining was performed as previously described^22^. After adipogenic differentiation, all media was removed and cells were fixed in 10% formalin for 5 min at room temperature. Formalin was replaced with the same volume of fresh formalin and incubated for 1 h. After removing the formalin, cells were washed with distilled water and incubated with 60% isopropanol for 5 min. The wells were dried completely and then stained with Oil red O working solution (60%, O1391, Sigma-Aldrich) for 10 minutes at room temperature. Oil red O was then removed and the wells immediately washed with distilled water three or four times. Red-stained lipid droplets were confirmed with a microscope. The stained lipid droplets were destained and quantified through absorbance measurements using a plate reader.

### Measurement of cell FFA

Primary adipocytes (2 × 10^5^) obtained from WT and RFP KO male mice were washed three times with PBS. Isoproterenol (ISP) was then added for 4 h to release FFA, and the cell supernatants were collected. FFA concentration was measured using a FFA assay kit (Abcam).

### Luciferase assay

The AP2 promoter plasmid, containing the luciferase-coding sequence under the transcriptional control of a 5400-bp-long fragment having both the enhancer and promoter elements of AP2 gene promoter, was further subcloned into a promoterless pGL3-Basic vector^23^. The PPAR-γ responsive element (PPRE) reporter was generated by inserting three copies of the DR1 sequence (TTCTGACCTATGACCTGG) into the pGL2-based basal reporter plasmid pTK-luc^24^. The luciferase assay was performed as previously described^21^. Next, 3T3-L1 cells were seeded into 24-well plates. When the cell confluency reached around 70–80%, cells were cotransfected with either 100 ng of empty pGL3UC vector or pGL3UC vector with AP2, along with si-RFP or RFP using Lipofectamine 3000. This method was repeated for the PPRE promoter. Luciferase and galactosidase activities were measured 48 h after transfection with the dual luciferase reporter assay system (Promega) following the manufacturer’s protocol. Luciferase activity was normalized by β-galactosidase luciferase activity.

### PLA

Protein interactions were assessed using a Duolink in situ red starter proximity ligation assay (PLA) kit (Sigma-Aldrich) following the manufacturer’s protocol. Cells were fixed with 4% paraformaldehyde, permeabilized with 0.1% Triton X-100 in 1× PBS and blocked with Duolink blocking buffer in a humidified chamber at 37 °C for 1 h. The cells were incubated with anti-RFP and anti-PPAR-γ antibodies overnight at 4 °C, followed by treatment with Duolink PLA probes (anti-rabbit Minus and anti-mouse Plus) for 1 h in a humidified chamber at 37 °C. After washing, PLA signals were generated through a ligation reaction for 30 min, followed by amplification for 90 min, both conducted in a a humidified chamber at 37 °C. Slides were mounted with ProLong Diamond Antifade Mountant with DAPI (Invitrogen) and images were captured using a Zeiss LSM880 confocal laser scanning microscope (Carl Zeiss).

### Chromatin immunoprecipitation

Chromatin immunoprecipitation assays were performed by using an EpiQuik chromatin immunoprecipitation kit (EpigenTek) according to the manufacturer’s instructions. Briefly, 3T3-L1 and HEK293T cells were treated with 1% formaldehyde for 10 min for crosslinking between proteins and DNA and sonicated to shear the chromatin into fragments of <500 bp. The chromatin complex was immunoprecipitated with anti-PPAR-γ or anti-HA, while the negative control was immunoprecipitated with nonimmunized IgG. The DNA fragments were detected by PCR. The primers used for PCR are provided in Supplementary Table 1.

### Electrophoretic mobility shift assays

*pCS4-HA-PPAR-γ* (HA−PPAR-γ) and *pFlag-CMV2-RFP* (Flag−RFP) were separately transfected to HEK293T cells. The lysates of HA−PPAR-γ-transfected cells were mixed with Flag−RFP-transfected cell lysates or mock-transfected cell lysates and subsequently incubated with biotinylated double-stranded oligonucleotides of PPRE present in the AP2 promoter for the gel shift assay. Anti-HA antibody was employed to demonstrate the supershift of PPAR-γ.

### Statistical analysis

Statistical analyses were conducted using IBM SPSS Statistics 26 (IBM) and GraphPad Prism software version 8. Data normality was assessed using the Shapiro−Wilk test. For data confirmed as normally distributed, parametric tests were applied, including a two-tailed Student’s *t*-test or analysis of variance (ANOVA) with subsequent post hoc analysis, depending on the number of groups (two or more). For comparisons involving more than two groups, one-way or two-way ANOVA was used, and variance homogeneity was tested with Levene’s test. For post hoc tests, Tukey’s honestly significant difference test or Bonferroni’s test was used for multiple comparisons with equal variance, and Dunnett’s T3 test or Šidák’s test for comparisons in unequal variance. For non-normally distributed data, as determined by the Shapiro−Wilk test, nonparametric methods were employed: the Kruskal−Wallis test with a Bonferroni adjustment or the Mann−Whitney *U* test, depending on the number of groups being compared. Statistical significance was confirmed for a *P* value of <0.05, and all *P* values are indicated in the corresponding figure legends.

## Results

### The expression of RFP is elevated in obesity

**Fig. 1:**
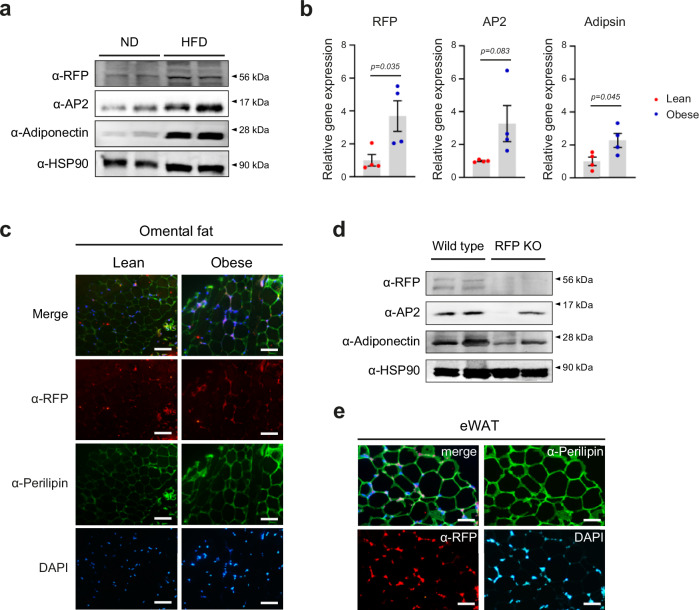
RFP expression is elevated in obese condition. **a** Western blot analysis of eWAT from WT mice after ND or HFD feeding. **b** qPCR analysis of RFP and adipogenic marker (AP2 and Adipsin) expression in autopsied fat tissue from obese human subjects. All data are presented as mean ± s.e.m. Statistical analysis was performed using unpaired Student’s *t*-tests. **c** RFP expression of omental fat tissue from lean and obese individuals. Scale bar, 100 μm. **d** Western blot analysis of adipose tissue from WT and RFP KO mice. **e** Immunofluorescence staining of eWAT from HFD-fed mice. Scale bar, 50 μm.

To investigate the role of RFP in lipid metabolism and its contribution to the homeostasis in skeletal muscle and in adipose tissue, we first investigated changes in RFP expression in HFD-fed WT mice. HFD administration substantial increased the protein levels of adipogenic markers, including AP2 and adiponectin in epididymal adipose tissue (Fig. 1a, rows two and three). Notably, RFP protein levels were also elevated in response to HFD (Fig. 1a, first row). To extend our findings to human subjects, we investigated the expression of RFP, along with adipogenic markers (AP2 and Adipsin) within human omental specimens. RFP and adipogenic marker expression showed an increase in obese subjects (Fig. 1b). Furthermore, immunofluorescent analysis revealed that RFP was co-localized with nuclei in the periphery of the tissue and the expression was increased in the omentum samples from obese individual compared with those from lean individuals obtained from cadaveric samples (Fig. 1c).

We next investigated the phenotype of RFP KO mouse. Analysis of adipose tissue obtained from RFP KO mice revealed a dramatic downregulation of adipogenesis-related proteins, such as AP2 and adiponectin (Fig. 1d). To further characterize the function of RFP, we examined its subcellular localization in adipose tissue of HFD-fed mice using immunofluorescence staining. RFP is predominantly localized in the nucleus in the periphery of the adipocytes, indicating potential roles within the nucleus (Fig. 1e). Taken together, these findings imply that RFP functions as a novel regulator of lipid metabolism and may play a critical role in lipid metabolic disorders.

### RFP deficiency protects against HFD-induced obesity

**Fig. 2:**
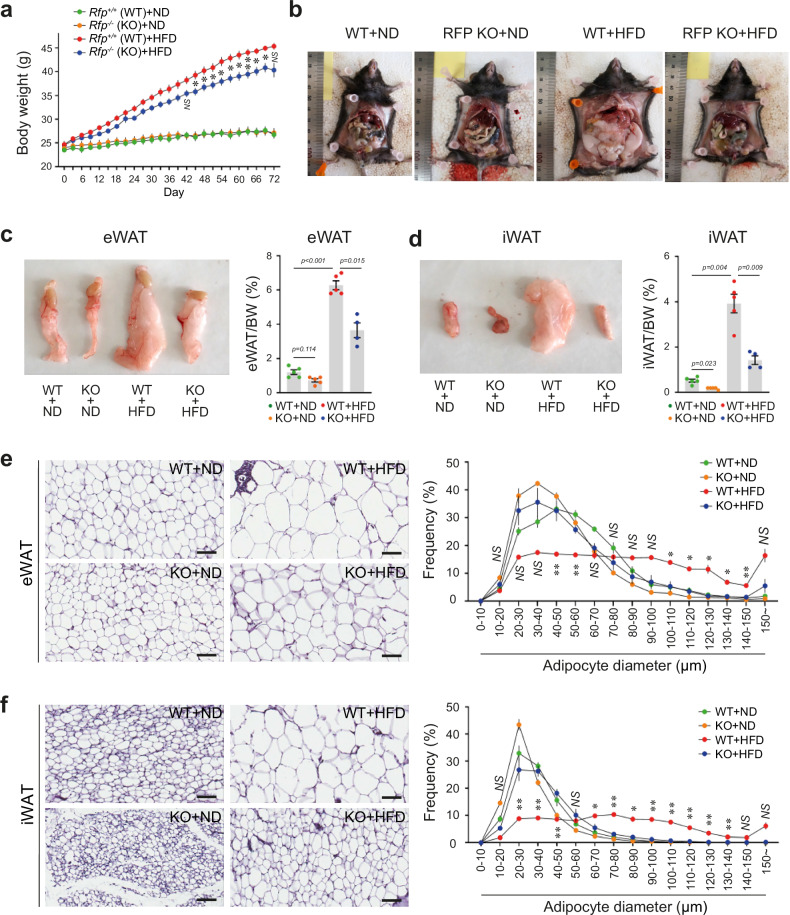
RFP deficiency protects against HFD-induced obesity in male mice. **a**, **b** Body weight (**a**) and fat mass (**b**) in WT and RFP KO mice after ND or HFD feeding. *n* = 5. Statistical analysis was performed using one-way ANOVA with post hoc Tukey’s test. **c**, **d** Weights of eWAT (**c**) and iWAT (**d**). *n* = 5. Statistical analysis was performed using one-way ANOVA with post hoc Dunnett’s T3 test. **e**, **f** H&E-stained sections of eWAT (**e**) and iWAT (**f**). Scale bar, 100 μm. *n* = 4. Statistical analysis was performed using a one-way ANOVA with post hoc Dunnett’s T3 test. All data are presented as mean ± s.e.m.

HFD feeding led to excessive accumulation of adipose tissue through increased energy intake, triggering obesity-associated metabolic disorders^25,26^. To investigate whether RFP deficiency affects HFD-induced obesity, we challenged both WT and RFP KO mice with HFD and monitored their metabolic responses. Notably, while WT mice developed significant increases in body weight and notable increases in fat mass upon HFD feeding, RFP KO mice exhibited remarkable resistance to HFD-induced weight gain and fat accumulation (Fig. 2a, b). Food intake did not differ significantly between WT and RFP KO mice (Supplementary Fig. 1a).

Adipose tissue regulates insulin sensitivity and energy balance; white adipose tissue (WAT) stores excess energy, while brown adipose tissue (BAT) is responsible for thermogenesis^27^. WAT dysfunction in obesity contributes to metabolic disorders such as type 2 diabetes^27,28^. In mice, eWAT is analogous to human visceral fat, while inguinal WAT (iWAT) is analogous to human subcutaneous fat^29^. Both eWAT and iWAT weights were significantly lower in HFD-fed RFP KO mice compared with WT controls (Fig. 2c, d). BAT weight showed no significant difference between WT and RFP KO mice (Supplementary Fig. 1b). While HFD increased the expression of adipose-related proteins such as AP2 and adiponectin in WT mice, these changes were markedly attenuated in RFP KO mice (Supplementary Fig. 1c). Histological analysis using H&E staining further demonstrated that RFP deficiency protected against HFD-induced adipocyte hypertrophy (Fig. 2e, f). The graphs in Fig. 2e, f show the frequency distribution of adipocyte diameters. WT mice (green dots) predominantly exhibit adipocytes in the ~40−50 μm range. In contrast, HFD feeding (red dots) induces a shift toward larger adipocytes, particularly in the ~80−140 μm range, indicating hypertrophy. This hypertrophic shift is mitigated in RFP-deficient mice (blue dots). Quantitative analysis was consistent with these findings, showing significantly smaller adipocytes in RFP KO mice (Supplementary Fig. 1d).

Subsequently, we examined the expression of adipogenic and lipogenic genes in eWAT obtained from HFD-treated WT and RFP KO mice. While HFD treatment upregulated the expression of all genes examined, this increase was significantly attenuated in eWAT from RFP KO mice compared with WT controls (Supplementary Fig. 2a, b).

### RFP deficiency improves glucose and lipid metabolism in HFD-fed mice

**Fig. 3:**
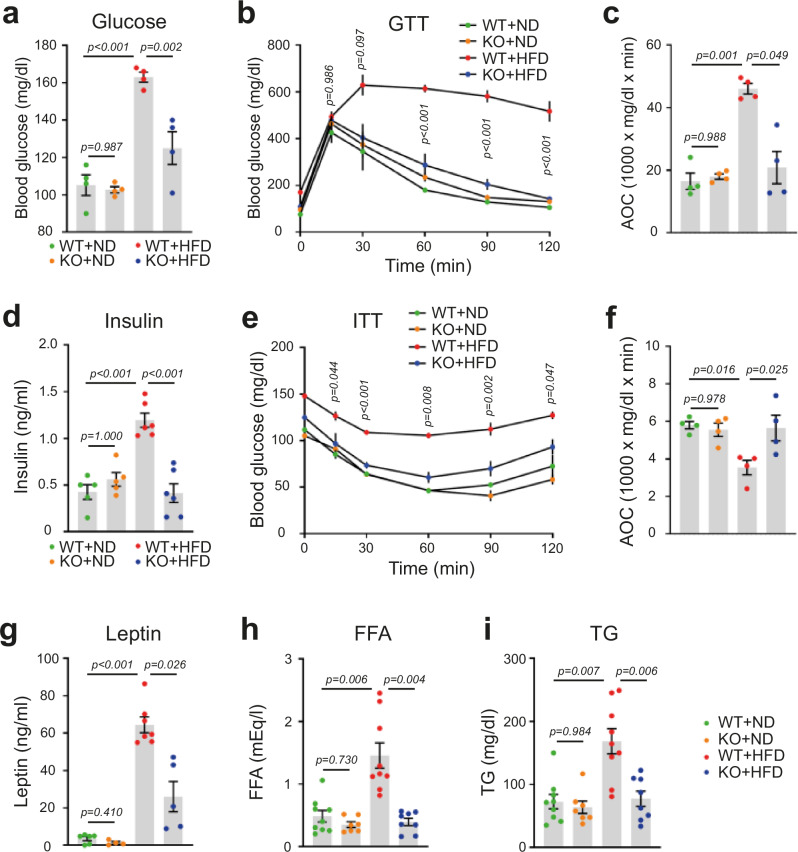
RFP deficiency improves glucose and lipid metabolism in HFD-fed male mice. **a**−**c** Blood glucose levels (**a**), GTT (**b**) and AOC (**c**) from the GTT on WT and RFP KO mice after ND or HFD feeding. *n* = 4. Statistical analysis was performed using one-way ANOVA with post hoc Tukey’s test (**a** and **b**) and Dunnett’s T3 test (**c**). **d**−**f** Blood insulin levels (**d**), ITT (**e**) and AOC (**f**) from ITT on WT and RFP KO mice after ND or HFD feeding. *n* = 4~6. Statistical analysis was performed using one-way ANOVA with post hoc Tukey’s test (**d** and **e**) and Bonferroni’s test (**f**). **g**−**i** Analysis of serum metabolic parameters including leptin (**g**), FFA (**h**) and TG (**i**) of WT and RFP KO mice after ND or HFD feeding. *n* = 4~9. Statistical analysis was performed using one-way ANOVA with post hoc Dunnett’s T3 test (**g** and **h**) and Bonferroni’s test (**i**). All data are presented as mean ± s.e.m.

HFD feeding impairs lipid and glucose homeostasis by disrupting insulin signaling pathways, leading to a decrease in glucose uptake and an increase in insulin resistance, thereby compromising overall metabolic health^30,31^. To assess HFD-induced metabolic dysfunction, we performed a glucose tolerance test (GTT) and insulin tolerance test (ITT). While HFD impaired glucose tolerance in WT mice (red dots), glucose metabolism was significantly improved in RFP KO mice fed with HFD (Fig. 3a–c, blue dots). Furthermore, RFP KO mice maintained normal insulin levels under HFD conditions (Fig. 3d−f, blue dots), whereas WT mice developed hyperinsulinemia (Fig. 3d, red dots). HFD-induced insulin resistance observed in WT mice was significantly attenuated in RFP KO mice (Fig. 3e, f).

Adipocyte-derived leptin plays an important role in the regulation of appetite and energy homeostasis, while circulating TG serves as the primary form of lipid transport for energy storage^32^. Dysregulation of leptin signaling (leptin resistance) and increased serum TG levels are associated with obesity-related disorders and insulin resistance^33,34^. RFP KO mice maintained lower levels of leptin, FFA and TG compared with WT mice (Fig. 3g–i). FFAs are primarily released into the bloodstream through adipose tissue lipolysis. Despite reduced fat accumulation, RFP KO mice showed minimal increases in serum FFA levels compared with the ND group. This may be due to their significantly lower total adipose tissue mass under HFD condition.

To further investigate the attenuated metabolic phenotypes, such as hyperglycemia and increased insulin resistance, in RFP KO mice from an energy metabolism perspective, we performed indirect calorimetry. Our analysis revealed that energy expenditure was increased in RFP KO-HFD mice (Supplementary Fig. 3a); however, energy intake, activity and heat production, which can influence energy expenditure, showed no significant differences compared with WT-HFD controls (Supplementary Fig. 3b−d). Collectively, these findings demonstrate that RFP deficiency robustly improves systemic glucose and lipid metabolism in HFD-fed mice, at least partially by enhancing energy expenditure.

### Adipocyte-specific RFP deficiency attenuates HFD-induced obesity

Our findings from global RFP KO mice demonstrated protection against HFD-induced metabolic dysfunction. However, metabolic homeostasis is regulated by complex interorgan communications involving multiple metabolically active tissues, including adipose tissue, liver, skeletal muscle and intestine^35,36^. Given that these organs can independently or cooperatively influence systemic metabolism and body weight regulation, it was crucial to determine whether the observed metabolic improvements in RFP KO mice were primarily mediated by adipose tissue or other metabolic organs. To address this question, we generated adipocyte-specific RFP KO (RFP cKO) mice using the Adipoq-Cre system (Supplementary Fig. 4a, b).

**Fig. 4:**
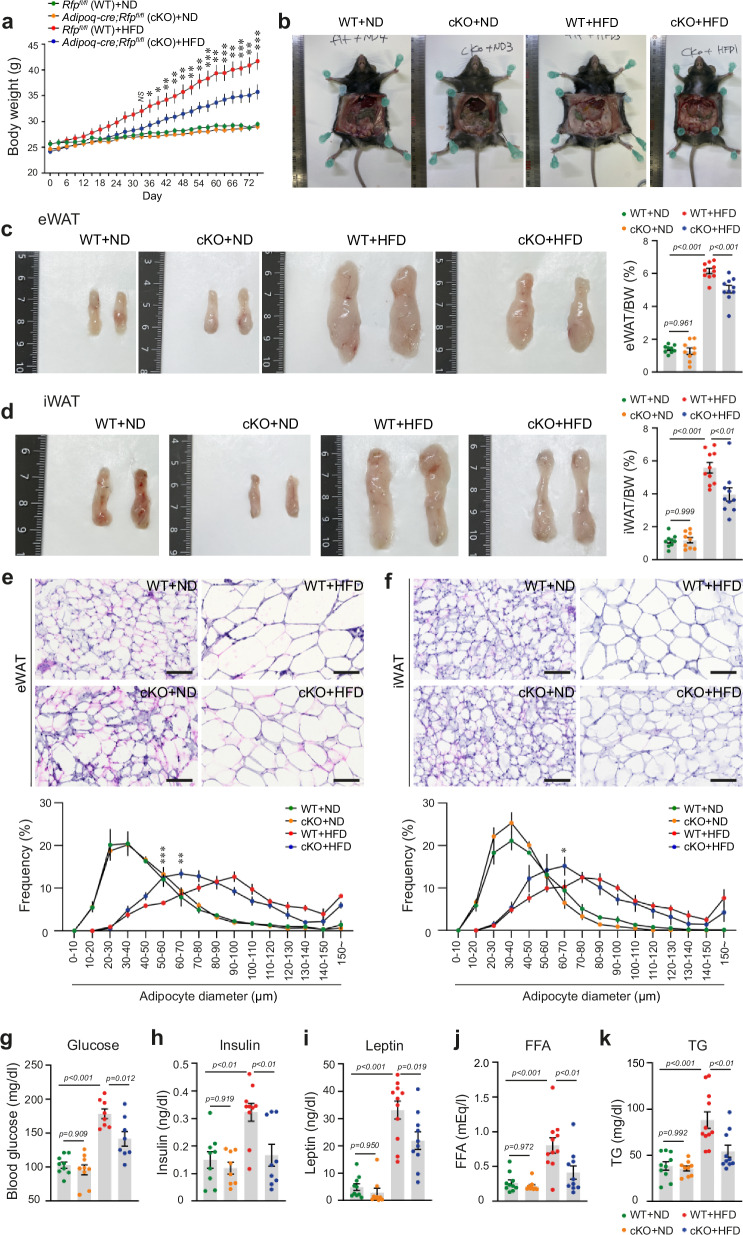
Adipocyte-specific RFP deficiency attenuates HFD-induced obesity in male mice. **a**−**d** Body weight (**a**), adipose tissue morphology (**b**), and adipose tissue mass (eWAT (**c**) and iWAT (**d**)) of RFP flox (WT) and RFP cKO mice after ND or HFD feeding. *n* = 9~10. Statistical analysis was performed using two-way ANOVA with post hoc Tukey’s test (**a**) and one-way ANOVA with post hoc Tukey’s test (**c** and **d**). **e**, **f** H&E-stained sections of adipose tissue (eWAT (**e**) and iWAT (**f**)) in WT and RFP cKO mice after ND or HFD feeding. Scale bar, 100 μm. *n* = 4. Statistical analysis was performed using two-way ANOVA with post hoc Tukey’s test. **g**−**k** Analysis of metabolic parameters (glucose (**g**), insulin (**h**), leptin (**i**), FFA (**j**) and TG (**k**)) of WT and RFP cKO mice after ND or HFD feeding. *n* = 8~11. Statistical analysis was performed using one-way ANOVA with post hoc Tukey’s test. All data are presented as mean ± s.e.m.

RFP cKO mice showed overall similar metabolic phenotypes to global RFP KO mice, particularly in terms of resistance to diet-induced obesity; when challenged with HFD, RFP cKO mice exhibited significant resistance to weight gain and adipose tissue accumulation compared with their control littermates (Fig. 4a–d), despite no significant differences in food intake or BAT weight (Supplementary Fig. 4c, d). Thus, adipocyte-specific deletion of RFP recapitulated the resistance to weight gain observed in whole-body RFP KO mice. Histological analysis of adipose tissue sections further revealed attenuated adipocyte hypertrophy in HFD-fed RFP cKO mice (Fig. 4e, f). Moreover, RFP cKO mice showed improved metabolic parameters, including reduced levels of serum glucose, insulin, leptin, FFA and TG (Fig. 4g–k). Furthermore, indirect calorimetry revealed that RFP cKO mice also exhibited increased energy expenditure compared with their control littermates (Supplementary Fig. 5a), without significant differences in food intake, activity or heat production (Supplementary Fig. 5b−d). Importantly, RFP cKO mice showed similar metabolic improvements to global RFP KO mice, suggesting that adipose tissue, rather than other metabolically active organs such as liver or skeletal muscle, is the primary mediator of RFP’s effects on systemic metabolism. On the basis of these findings, we focused our subsequent mechanistic studies on adipocytes.

### RFP deficiency does not alter adipocyte lipolysis

Our in vivo studies revealed reduced serum FFA levels in RFP KO mice following HFD challenge (Fig. 3h). Since FFAs are primarily released into circulation through adipose tissue lipolysis^37^, we initially hypothesized that RFP might regulate lipolysis. However, the reduction of serum FFA levels could also result from the decrease in total adipose tissue mass rather than impairment of lipolytic activity in individual adipocytes. To distinguish between these possibilities, we conducted both in vivo and in vitro analyses of lipolysis.

**Fig. 5:**
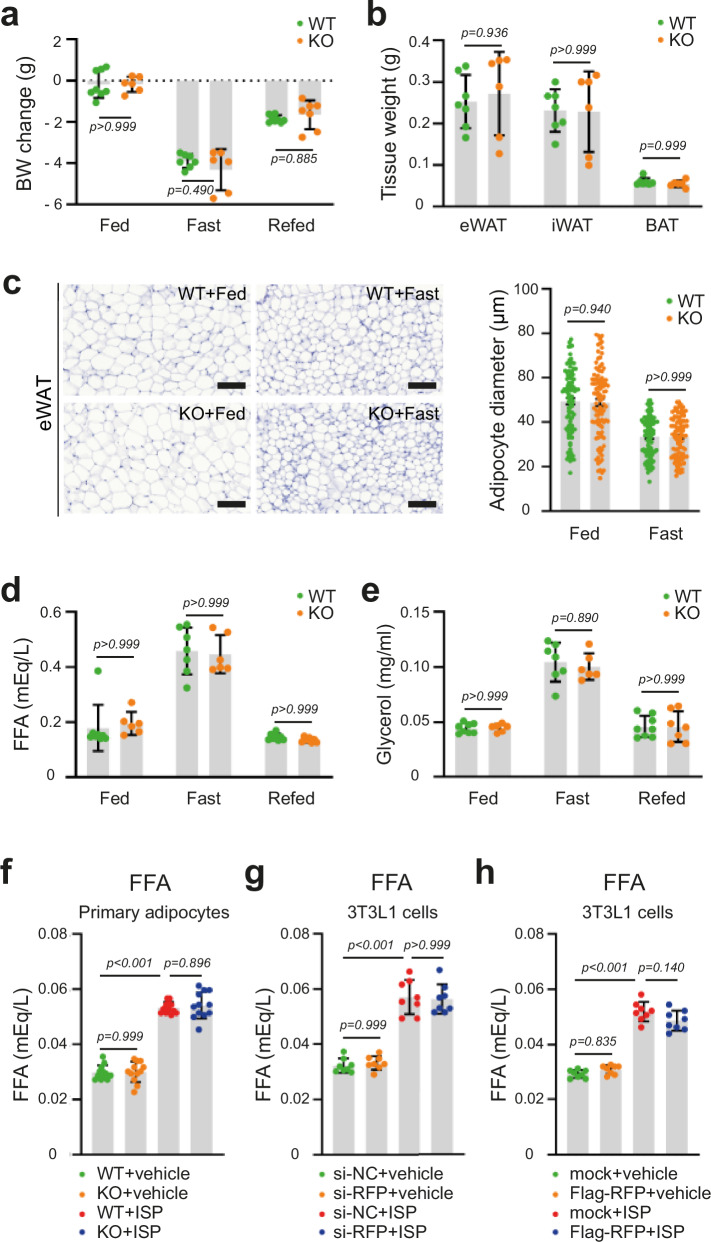
RFP deficiency does not affect lipolytic activity in adipocytes. **a**, **b** Body weight (BW) (**a**) and adipose tissue (eWAT, iWAT and BAT) mass (**b**) in WT and RFP KO mice under feed−fast−refeed conditions. *n* = 6~8. Statistical analysis was performed using two-way ANOVA with post hoc Šidák’s test. **c** Histological analysis of eWAT in fed and fast groups. Scale bar, 100 μm. *n* = 100. Two-way ANOVA with post hoc Šidák’s test. **d**, **e** Serum FFA (**d**) and glycerol (**e**) levels in WT and RFP KO mice of fed, fast and refed groups. *n* = 6~8. Statistical analysis was performed using two-way ANOVA with post hoc Sidak’s test. **f** ISP-induced FFA release in primary adipocytes isolated from eWAT of WT and RFP KO mice. *n* = 12. Statistical analysis was performed using one-way ANOVA with post hoc Tukey’s test. **g**, **h** ISP-induced FFA release in 3T3-L1 adipocytes with RFP knockdown (**g**) or overexpression (**h**). *n* = 8. Statistical analysis was performed using two-way ANOVA with post hoc Šidák’s test. All data are presented as mean ± s.e.m.

Next, we examined fasting-induced lipolysis in WT and RFP KO mice using a feed−fast−refeed protocol (24-h fasting followed by 6-h refeeding). Body weights and adipose tissue masses (eWAT, iWAT and BAT) showed similar changes between WT and RFP KO mice across all feeding conditions (Fig. 5a, b). Histological analysis of eWAT showed similar fasting-induced reduction in adipocyte size between two groups (Fig. 5c). Moreover, fasting-induced increases in serum FFA and glycerol levels, and their normalization upon refeeding, were similar between WT and RFP KO mice (Fig. 5d, e).

To directly assess adipocyte-autonomous lipolytic capacity, we isolated primary adipocytes from the eWAT of WT and RFP KO mice and treated them with ISP to induce FFA release. FFA release from equal numbers of primary adipocytes showed no significant differences between WT and KO mice (Fig. 5f). We next utilized 3T3-L1 cells and tested two types of siRNAs with different sequences in 3T3-L1 cells. Both were confirmed to effectively knock down RFP (Supplementary Fig. 6). Consistent with previous findings, neither RFP knockdown nor overexpression of RFP in 3T3-L1 adipocytes affected ISP-induced FFA release (Fig. 5g, h). Collectively, these results indicate that the reduced serum FFA levels observed in RFP KO mice under HFD conditions probably reflect the decrease in total adipose tissue mass rather than impaired lipolysis in individual adipocyte. These findings suggest that RFP regulates fat accumulation through mechanisms independent of lipolysis.

### Transcriptome analysis reveals altered adipogenesis and lipid metabolism in RFP KO mice after HFD feeding

**Fig. 6:**
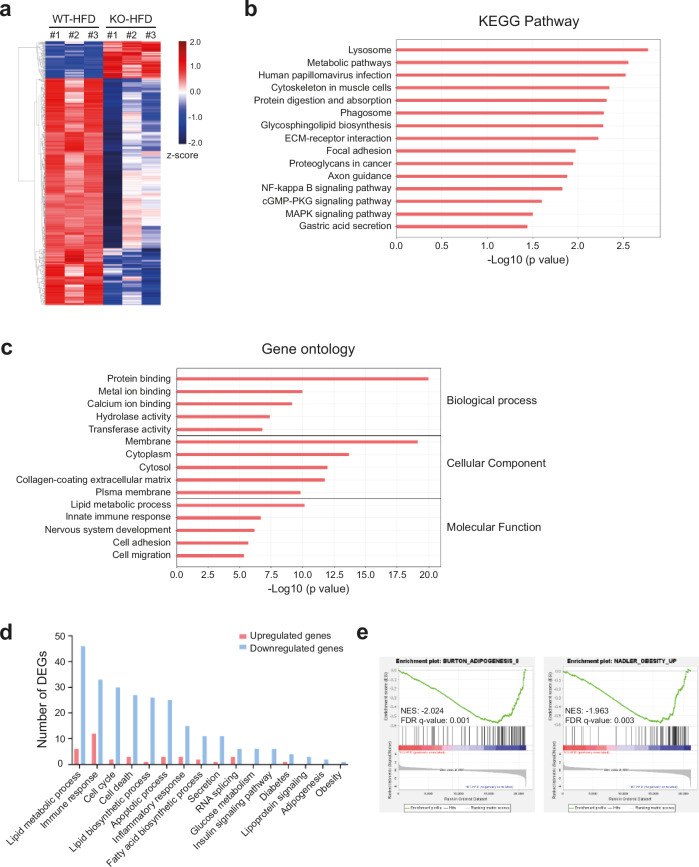
Transcriptome analysis reveals altered adipogenesis and lipid metabolism in RFP KO male mice after HFD feeding. DEGs between WT-HFD and RFP KO-HFD were identified based on criteria of *P* value ≤0.05 and |log_2_ fold change| ≥2. **a** A heat map of DEGs between WT-HFD and RFP KO-HFD groups. **b** KEGG pathway analysis of DEGs. **c** GO enrichment analysis categorizing DEGs into biological processes, cellular components and molecular functions. **d** DEG expression patterns in several GO category classifications. **e** GSEA showing enrichment of adipogenesis and obesity-related pathways. NES represents the degree to which a gene set is overrepresented at the top or bottom of the ranked gene list, normalized across all analyzed gene sets. FDR indicates the estimated probability that the observed enrichment is a false positive. Both gene sets are significantly downregulated in RFP KO-HFD. FDR, false discovery rate; NES, normalized enrichment score.

To determine how RFP KO mice were protected from metabolic disorder, we analyzed the transcriptome from isolated eWAT after HFD feeding. First, we obtained statistically significant and differentially expressed genes (DEGs) between HFD-fed WT or RFP KO mice from RNA-sequencing. DEGs were identified based on the criteria of *P* value ≤0.05 and |log_2_ fold change| ≥2. Heat map analysis of the DEGs revealed distinct gene expression profiles between the WT-HFD and RFP KO-HFD groups. Genes with increased expression (Fig. 6a, red) were predominantly observed in WT-HFD, while genes with decreased expression (Fig. 6a, blue) were prominent in KO-HFD. This pattern suggests a substantial shift in the transcriptional landscape driven by KO-HFD treatment (Fig. 6a). To acquire comprehensive data on the functional enrichment of pathways, DEGs were further analyzed by ExDEGA. KEGG pathway analysis indicated enrichment in metabolic pathways, lysosome-related processes, and cytoskeletal regulation in muscle cells (Fig. 6b). Gene ontology (GO) is subdivided into three categories: biological process, cellular component and molecular function. GO analysis showed DEGs were mainly enriched in processes related to protein binding in the biological process group, membrane in the cellular component group and lipid metabolic process in the molecular function group (Fig. 6c). DEGs associated with lipid metabolic processes exhibited a higher number of downregulated genes in the RFP KO-HFD group compared with the WT-HFD group, as indicated by the blue bars. Specifically, lipid-related pathways, including fatty acid beta-oxidation and lipid transport, were significantly suppressed in the RFO KO-HFD group (Fig. 6d). Gene Set Enrichment Analysis (GSEA) using a curated gene set database, revealed significant association with adipogenesis and obesity (Fig. 6e). On the basis of these findings, we hypothesized that attenuation of the obesity phenotype observed in RFP KO mice is associated with alteration in adipogenesis and lipid metabolism.

### RFP deficiency suppresses adipogenic differentiation

**Fig. 7:**
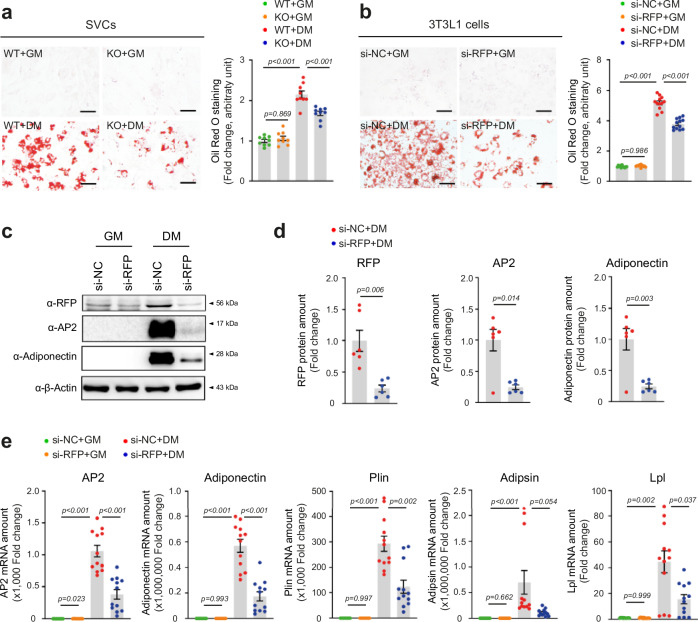
RFP knockdown suppresses adipogenic differentiation. **a**, **b** Oil red O staining of adipocytes derived from primary SVCs of WT and RFP KO male mice (**a**) and RFP-knockdown 3T3-L1 cells (**b**). *n* = 9. Statistical analysis was performed using one-way ANOVA with post hoc Tukey’s test. **c** Western blot analysis of adipogenic markers of RFP-knockdown 3T3-L1 adipocytes. **d** Densitometric analysis of protein expression of RFP, AP2 and adiponectin in RFP-deficient 3T3-L1 adipocytes. *n* = 6. Statistical analysis was performed using unpaired Student’s *t*-test. **e** RT−qPCR analysis of adipogenic genes, including AP2, adiponectin, Plin, adipsin and Lpl in RFP-depleted 3T3-L1 cells compared with controls. *n* = 12. Statistical analysis was performed using one-way ANOVA with post hoc Dunnett’s T3 test. All data are presented as mean ± s.e.m.

To elucidate the molecular mechanisms underlying RFP-mediated regulation of adipose tissue accumulation, we investigated the role of RFP in adipocyte differentiation using both primary cells and an established cell line. Primary SVCs were isolated from WAT of WT and RFP KO mice. RFP was knocked down in 3T3-L1 cells before inducing differentiation. Both SVCs and 3T3-L1 preadipocytes were subjected to adipogenic differentiation for 8 days after reaching confluence. Oil red O staining demonstrated that lipid accumulation was significantly reduced in both RFP-deficient primary SVCs and RFP-knockdown 3T3-L1 cells compared with their respective controls (Fig. 7a, b). At the molecular level, RFP knockdown markedly suppressed adipocyte differentiation, accompanied with significant attenuation of the expression of adipogenic genes, including AP2 and adiponectin. The protein expression was quantified by densitometric analysis (Fig. 7c, d). Consistent with the protein expression data, RT−qPCR analysis demonstrated significant downregulation of adipogenic genes, including AP2, adiponectin, perilipin (Plin), adipsin and lipoprotein lipase (Lpl) in RFP-depleted cells (Fig. 7e). These findings strongly suggest that RFP functions as a positive regulator of adipogenesis by modulating the expression of key adipogenic transcription factors and their downstream target genes.

### RFP physically interacts with PPAR-γ and enhances its transcriptional activity

The marked reduction in adipogenic gene expression following RFP depletion led us to investigate potential molecular mechanisms underlying RFP-mediated regulation of adipogenesis. The adipogenesis process can be governed by many critical transcription factors. Given that PPAR-γ orchestrates adipogenic differentiation through complex interactions with various transcription factors^38^ such as CCAAT enhancer-binding protein (C/EBP) family (C/EBPα, C/EBPβ and C/EBPδ)^39,40^ and sterol regulatory element binding protein 1c (SREBP1c)^40^, we hypothesized that RFP might regulate adipogenesis through PPAR-γ-dependent mechanisms.

**Fig. 8:**
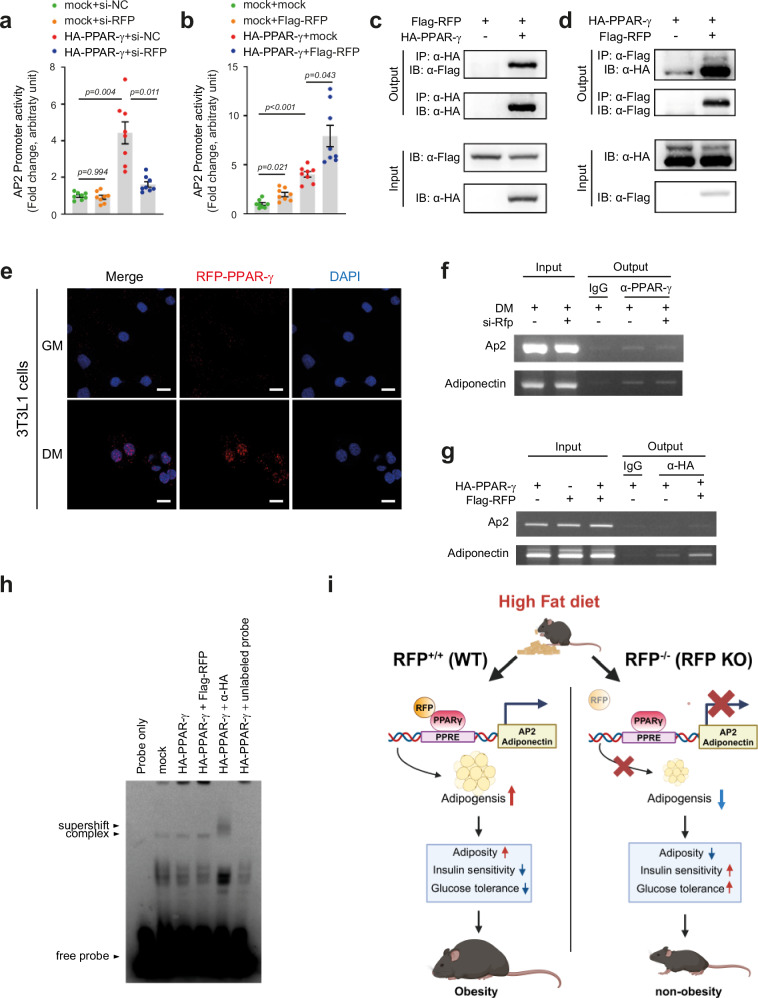
RFP physically interacts with PPAR-γ and enhances its transcriptional activity. **a**, **b** Effect of RFP knockdown (**a**) or overexpression (**b**) on AP2 promoter activity in 3T3-L1 cells. *n* = 8. Statistical analysis was performed using one-way ANOVA with post hoc Dunnett’s T3 test. **c**, **d** Immunoprecipitation (IP) experiments in HEK293T cells expressing Flag−RFP and HA−PPAR-γ reveal robust protein−protein interactions, which were further confirmed by reciprocal immunoprecipitation. IP with anti-HA followed by immunoblotting with anti-Flag confirmed the interaction (**c**), while IP with anti-Flag followed by detection with anti-HA yielded consistent results (**d**). **e** In situ PLA in differentiated 3T3-L1 adipocytes. Scale bar, 20 μm. **f**, **g** The effect of RFP knockdown (**f**) or overexpression (**g**) on PPAR-γ binding to PPRE in 3T3-L1 cells. **h** The electrophoretic mobility shift assay demonstrates that RFP promotes PPAR-γ binding to PPRE sequences within the AP2 promoter. **i** A Schematic diagram showing that the metabolic improvements observed in RFP-deficient mice under HFD conditions result from attenuated PPAR-γ-dependent adipogenic programming. Panel **i** was created with BioRender.com. All data are presented as mean ± s.e.m.

To test this hypothesis, we first examined whether RFP modulates PPAR-γ-dependent transcriptional activity. In reporter assays, RFP knockdown significantly attenuated PPAR-γ-mediated activation of the AP2 promoter in 3T3-L1 cells, while RFP overexpression enhanced PPRE promoter activity (Fig. 8a,b). Similar effects were observed using a synthetic PPRE promoter containing DR1 repeats (Supplementary Fig. 7a).

We next investigated physical interactions between RFP and PPAR-γ. Coimmunoprecipitation experiments in 293T cells expressing Flag−RFP and HA−PPAR-γ revealed robust protein−protein interactions, which were confirmed by reciprocal immunoprecipitation (Fig. 8c, d). The physiological relevance of this interaction was validated using in situ PLAs, which demonstrated increased nuclear RFP−PPAR-γ complexes in differentiated compared with undifferentiated 3T3-L1 adipocytes (Fig. 8e). We generated mutant constructs to identify the domain responsible for RFP binding to PPAR-γ, and our results suggest that the coiled-coil domain of RFP plays a critical role in mediating its interaction with PPAR-γ (Supplementary Fig. 7b−d).

Functionally, RFP knockdown suppressed PPAR-γ binding to PPRE and reduced expression of PPAR-γ target genes including AP2 and adiponectin (Fig. 8f). Conversely, RFP overexpression enhanced PPAR-γ binding to PPRE and increased target gene expression (Fig. 8g). Electrophoretic mobility shift assays further confirmed that RFP promotes PPAR-γ binding to PPRE sequences within the AP2 promoter, as evidenced by enhanced complex formation and successful supershift with anti-HA antibody (Fig. 8h). Collectively, our in vivo and in vitro findings demonstrate that RFP functions as a positive regulator of adipogenesis by physically interacting with PPAR-γ and enhancing its transcriptional activity at target promoters.

## Discussion

In this study, we discovered a novel function of RFP as a critical regulator of adipogenesis and systemic metabolism. We demonstrated that RFP expression is elevated in the adipose tissue of both HFD-fed mice and obese human subjects. Genetic deletion of RFP, whether globally or specifically in adipocytes, significantly attenuated HFD-induced obesity and associated metabolic dysfunction, including reduced adiposity, improved glucose tolerance and enhanced insulin sensitivity (Fig. 8i). These findings underscore the pivotal role of RFP in adipose tissue expansion and systemic metabolic regulation.

One of the notable findings in this study is that global RFP KO and adipocyte-specific RFP KO mice exhibit a similar pattern of protection against HFD-induced obesity, albeit with some differences in the degree of metabolic improvement. This suggests that the beneficial effects of RFP deficiency on metabolic disorders are primarily mediated by adipocytes, rather than other metabolically active tissues. These results support the hypothesis that adipocyte-specific mechanisms are central to the metabolic improvements observed in RFP-deficient mice. However, it requires further examination whether RFP plays additional roles in non-adipose tissues, as the complex interorgan communication involved in metabolic regulation cannot be excluded^35,36^.

Mechanistically, our study demonstrates that RFP directly interacts with PPAR-γ and enhances its transcriptional activity, promoting adipocyte differentiation and the expression of key adipogenic genes. This interaction probably underlies the observed adipose tissue expansion in HFD-fed WT mice, whereas RFP deficiency attenuates this process. While PPAR-γ appears to be a major downstream target of RFP in adipocytes, it may not be the sole mediator of RFP’s metabolic effects. Given RFP’s known role as a multifunctional regulator of transcriptional activity and protein−protein interactions, it is plausible that RFP also influences other signaling pathways or transcriptional regulators, either directly or indirectly. Identifying additional targets of RFP will be critical for fully understanding its role in obesity and systemic metabolism^39–41^.

An intriguing aspect of RFP’s functionality is its role as a molecular switch that balances myogenic and adipogenic differentiation. Previous studies demonstrated that RFP negatively regulates myogenesis by promoting MyoD degradation and transcriptional repression^13^. In contrast, our present findings reveal RFP’s role as a positive regulator of adipogenesis, mediated through PPAR-γ activation. This dual regulatory mechanism underscores RFP’s critical role in determining cell fate between muscle and adipose lineages. The reciprocal relationship between muscle mass and adiposity is well documented, with increased adiposity often correlating with reduced muscle mass in various metabolic conditions^42^. For example, transcription factors such as IL-15, acylation-stimulating protein and adiponectin also contribute to maintaining the balance between these tissues^43^. Further understanding RFP’s dual function, particularly its ability to simultaneously suppress myogenic and promote adipogenic pathways, provides vital insights into the molecular basis of body composition regulation.

The observed elevation of RFP expression in both diet-induced obese mice and obese human subjects raises intriguing questions about its potential role in obesity pathogenesis. It is possible that increased RFP levels act as part of a maladaptive feedback mechanism, exacerbating adipose tissue expansion and systemic metabolic dysfunction through enhanced PPAR-γ activation. This mechanism may contribute to the progressive nature of obesity and its associated complications, such as insulin resistance and type 2 diabetes^44,45^. Furthermore, the reduced adiposity and improved glucose metabolism observed in RFP-deficient mice highlight RFP’s critical role in coordinating adipose tissue function with systemic energy homeostasis.

In our study, RFP deficiency improved metabolic disorders in HFD-induced obesity models by downregulating PPAR-γ target genes, leading to reduced fat accumulation and improved insulin sensitivity. The role of PPAR-γ in insulin resistance, however, is complex and context dependent. While its inhibition has been associated with exacerbating insulin resistance through increased fatty acid release and inflammatory responses^46,47^, selective inhibition can also enhance insulin sensitivity by limiting excessive fat accumulation^48,49^. Overexpression of PPAR-γ, on the other hand, often promotes excessive adiposity and metabolic dysfunction^48,50^. These findings suggest that the relationship between PPAR-γ activity and insulin resistance is highly nuanced, with its modulation yielding both positive and negative outcomes depending on the context. Our results align with the notion that reduced PPAR-γ activity in adipocytes can mitigate obesity-associated metabolic dysfunction and improve systemic insulin sensitivity.

While these findings enhance our understanding of PPAR-γ modulation, key questions remain. The upstream mechanisms regulating RFP expression in obesity and its role in other metabolic tissues, such as the liver, warrant further study. Given the liver’s central role in glucose and lipid homeostasis, liver-specific RFP functions may reveal additional insights into systemic metabolism. Furthermore, the long-term effects of RFP inhibition and its roles in immunity and cell proliferation should be carefully evaluated^7–9^.

Energy metabolism fundamentally differs between sexes, regulated by a complex interplay of sex chromosomes, hormones and other biological factors. Consequently, fluctuations in estrogen and other sex hormones throughout the estrous cycle can markedly influence metabolic parameters such as glucose tolerance, insulin sensitivity and body weight regulation^51,52^. Therefore, male mice have traditionally been widely used in metabolic studies to minimize this variability. Following this conventional approach, our experimental design also exclusively employed male mice. However, recent guidelines and best practices emphasize the importance of including both sexes or, at the very least, clearly stating the sex of animals used in preclinical studies^53^. Such emphasis is driven by increasing evidence that neglecting sex as a biological variable can lead to pronounced disparities in outcomes, as exemplified by sex-specific differences in drug pharmacokinetics and adverse drug reactions^54^. We acknowledge that this poses a limitation to the generalizability of our findings.

In conclusion, this study identifies RFP as a key regulator of adipose tissue expansion and metabolic regulation. While therapies directly targeting adipogenesis remain rare and more effective targets for obesity and metabolic disease are likely to exist, the role of RFP in balancing adipose and skeletal muscle metabolism presents a compelling mechanism of scientific interest. This understanding may not only shed light on the intricate pathways governing metabolic homeostasis but also guide the identification of more ideal therapeutic targets for obesity and related disorders. While the potential of targeting adipogenesis for therapeutic purposes requires further exploration, our findings provide important insights into the mechanisms underlying systemic metabolism. Future research should focus on uncovering additional RFP targets and clarifying its role in interorgan communication, thereby contributing to a deeper understanding of metabolic diseases and paving the way for potential therapeutic strategies.

## Supplementary information

Supplementary Information

## Data Availability

All data needed to evaluate the conclusions in the paper are present in the paper and/or the Supplementary Materials.

## References

[1] World Health Organization. *Obesity and Overweight. Fact Sheets: World Health Organization* (WHO, 2024).

[2] NCD Risk Factor Collaboration. Worldwide trends in underweight and obesity from 1990 to 2022: a pooled analysis of 3663 population-representative studies with 222 million children, adolescents, and adults. *Lancet* **403**, 1027–1050 (2024). 38432237 10.1016/S0140-6736(23)02750-2 PMC7615769

[3] Kim, D. S. & Scherer, P. E. Obesity, diabetes, and increased cancer progression. *Diabetes Metab. J.* **45**, 799–812 (2021). 34847640 10.4093/dmj.2021.0077 PMC8640143

[4] Baldelli, S. et al. The role of adipose tissue and nutrition in the regulation of adiponectin. *Nutrients* **16**, 2436 (2024). 10.3390/nu16152436 PMC11313710 39125318

[5] Zoumpoulidou, G. et al. Role of the tripartite motif protein 27 in cancer development. *J. Natl Cancer Inst.* **104**, 941–952 (2012). 22556269 10.1093/jnci/djs224

[6] Hatakeyama, S. TRIM family proteins: roles in autophagy, immunity, and carcinogenesis. *Trends Biochem. Sci.* **42**, 297–311 (2017). 28118948 10.1016/j.tibs.2017.01.002

[7] Ozato, K., Shin, D. M., Chang, T. H. & Morse, H. C. 3rd TRIM family proteins and their emerging roles in innate immunity. *Nat. Rev. Immunol.* **8**, 849–860 (2008). 18836477 10.1038/nri2413 PMC3433745

[8] Liu, S. et al. TRIM27 acts as an oncogene and regulates cell proliferation and metastasis in non-small cell lung cancer through SIX3-β-catenin signaling. *Aging* **12**, 25564–25580 (2020). 33264103 10.18632/aging.104163 PMC7803540

[9] Yu, C. et al. Emerging roles of TRIM27 in cancer and other human diseases. *Front. Cell Dev. Biol.* **10**, 1004429 (2022). 36200036 10.3389/fcell.2022.1004429 PMC9527303

[10] Yang, Y. et al. TRIM27 cooperates with STK38L to inhibit ULK1-mediated autophagy and promote tumorigenesis. *EMBO J.* **41**, e109777 (2022). 35670107 10.15252/embj.2021109777 PMC9289709

[11] Ahsan, N. et al. Multipronged regulation of autophagy and apoptosis: emerging role of TRIM proteins. *Cell Mol. Biol. Lett.* **29**, 13 (2024). 38225560 10.1186/s11658-023-00528-8 PMC10790450

[12] Kee, H. J. et al. Ret finger protein inhibits muscle differentiation by modulating serum response factor and enhancer of polycomb1. *Cell Death Differ.* **19**, 121–131 (2012). 21637294 10.1038/cdd.2011.72 PMC3252832

[13] Joung, H. et al. Ret finger protein mediates Pax7-induced ubiquitination of MyoD in skeletal muscle atrophy. *Cell Signal.* **26**, 2240–2248 (2014). 25025573 10.1016/j.cellsig.2014.07.006

[14] Vinue, A. & Gonzalez-Navarro, H. Glucose and insulin tolerance tests in the mouse. *Methods Mol. Biol.* **1339**, 247–254 (2015). 26445794 10.1007/978-1-4939-2929-0_17

[15] Virtue, S. & Vidal-Puig, A. GTTs and ITTs in mice: simple tests, complex answers. *Nat. Metab.* **3**, 883–886 (2021). 34117483 10.1038/s42255-021-00414-7

[16] Yu, H. C. et al. p21-activated kinase 4 counteracts PKA-dependent lipolysis by phosphorylating FABP4 and HSL. *Nat. Metab.* **6**, 94–112 (2024). 38216738 10.1038/s42255-023-00957-x

[17] Iwanaga, J. et al. Standardized statement for the ethical use of human cadaveric tissues in anatomy research papers: recommendations from Anatomical Journal Editors-in-Chief. *Clin. Anat.* **35**, 526–528 (2022). 35218594 10.1002/ca.23849

[18] Han, Y., Kim, C. Y., Cheong, H. & Lee, K. Y. Osterix represses adipogenesis by negatively regulating PPARγ transcriptional activity. *Sci. Rep.* **6**, 35655 (2016). 27752121 10.1038/srep35655 PMC5067693

[19] Ruan, H., Zarnowski, M. J., Cushman, S. W. & Lodish, H. F. Standard isolation of primary adipose cells from mouse epididymal fat pads induces inflammatory mediators and down-regulates adipocyte genes. *J. Biol. Chem.* **278**, 47585–47593 (2003). 12975378 10.1074/jbc.M305257200

[20] Zebisch, K., Voigt, V., Wabitsch, M. & Brandsch, M. Protocol for effective differentiation of 3T3-L1 cells to adipocytes. *Anal. Biochem* **425**, 88–90 (2012). 22425542 10.1016/j.ab.2012.03.005

[21] Kee, H. J. et al. Enhancer of polycomb1, a novel homeodomain only protein-binding partner, induces skeletal muscle differentiation. *J. Biol. Chem.* **282**, 7700–7709 (2007). 17192267 10.1074/jbc.M611198200

[22] Mehlem, A., Hagberg, C. E., Muhl, L., Eriksson, U. & Falkevall, A. Imaging of neutral lipids by oil red O for analyzing the metabolic status in health and disease. *Nat. Protoc.* **8**, 1149–1154 (2013). 23702831 10.1038/nprot.2013.055

[23] Rival, Y. et al. Human adipocyte fatty acid-binding protein (aP2) gene promoter-driven reporter assay discriminates nonlipogenic peroxisome proliferator-activated receptor gamma ligands. *J. Pharm. Exp. Ther.* **311**, 467–475 (2004). 10.1124/jpet.104.068254 15273253

[24] Kim, J. E. & Chen, J. regulation of peroxisome proliferator-activated receptor-gamma activity by mammalian target of rapamycin and amino acids in adipogenesis. *Diabetes* **53**, 2748–2756 (2004). 15504954 10.2337/diabetes.53.11.2748

[25] Reaven, G. M. Banting lecture 1988. Role of insulin resistance in human disease. *Diabetes* **37**, 1595–1607 (1988). 3056758 10.2337/diab.37.12.1595

[26] Grundy, S. M. Obesity, metabolic syndrome, and coronary atherosclerosis. *Circulation* **105**, 2696–2698 (2002). 12057978 10.1161/01.cir.0000020650.86137.84

[27] Rosen, E. D. & Spiegelman, B. M. Adipocytes as regulators of energy balance and glucose homeostasis. *Nature* **444**, 847–853 (2006). 17167472 10.1038/nature05483 PMC3212857

[28] Trayhurn, P. Endocrine and signalling role of adipose tissue: new perspectives on fat. *Acta Physiol. Scand.* **184**, 285–293 (2005). 16026420 10.1111/j.1365-201X.2005.01468.x

[29] Lee, M. J., Wu, Y. & Fried, S. K. Adipose tissue heterogeneity: implication of depot differences in adipose tissue for obesity complications. *Mol. Asp. Med* **34**, 1–11 (2013). 10.1016/j.mam.2012.10.001 PMC3549425 23068073

[30] Hotamisligil, G. S. Inflammation and metabolic disorders. *Nature* **444**, 860–867 (2006). 17167474 10.1038/nature05485

[31] Samuel, V. T. & Shulman, G. I. The pathogenesis of insulin resistance: integrating signaling pathways and substrate flux. *J. Clin. Invest.* **126**, 12–22 (2016). 26727229 10.1172/JCI77812 PMC4701542

[32] Havel, P. J. Update on adipocyte hormones: regulation of energy balance and carbohydrate/lipid metabolism. *Diabetes* **53**, S143–S151 (2004). 14749280 10.2337/diabetes.53.2007.s143

[33] Friedman, J. M. & Halaas, J. L. Leptin and the regulation of body weight in mammals. *Nature* **395**, 763–770 (1998). 9796811 10.1038/27376

[34] Walker, M. Obesity, insulin resistance, and its link to non-insulin-dependent diabetes mellitus. *Metabolism* **44**, 18–20 (1995). 7674911 10.1016/0026-0495(95)90314-3

[35] Liu, S., Alexander, R. K. & Lee, C. H. Lipid metabolites as metabolic messengers in inter-organ communication. *Trends Endocrinol. Metab.* **25**, 356–363 (2014). 24895003 10.1016/j.tem.2014.05.002 PMC4077945

[36] Priest, C. & Tontonoz, P. Inter-organ cross-talk in metabolic syndrome. *Nat. Metab.* **1**, 1177–1188 (2019). 32694672 10.1038/s42255-019-0145-5

[37] Coppack, S. W., Jensen, M. D. & Miles, J. M. In vivo regulation of lipolysis in humans. *J. Lipid Res* **35**, 177–193 (1994). 8169522

[38] Shao, X. et al. Peroxisome proliferator-activated receptor-gamma: master regulator of adipogenesis and obesity. *Curr. Stem Cell Res. Ther.* **11**, 282–289 (2016). 26018229 10.2174/1574888x10666150528144905

[39] Yeh, W. C., Cao, Z., Classon, M. & McKnight, S. L. Cascade regulation of terminal adipocyte differentiation by three members of the C/EBP family of leucine zipper proteins. *Genes Dev.* **9**, 168–181 (1995). 7531665 10.1101/gad.9.2.168

[40] Rosen, E. D., Walkey, C. J., Puigserver, P. & Spiegelman, B. M. Transcriptional regulation of adipogenesis. *Genes Dev.* **14**, 1293–1307 (2000). 10837022

[41] Zhang, H. X. et al. TRIM27 mediates STAT3 activation at retromer-positive structures to promote colitis and colitis-associated carcinogenesis. *Nat. Commun.* **9**, 3441 (2018). 30143645 10.1038/s41467-018-05796-z PMC6109048

[42] Di Iorgi, N., Rosol, M., Mittelman, S. D. & Gilsanz, V. Reciprocal relation between marrow adiposity and the amount of bone in the axial and appendicular skeleton of young adults. *J. Clin. Endocrinol. Metab.* **93**, 2281–2286 (2008). 18381577 10.1210/jc.2007-2691 PMC2435643

[43] Argiles, J. M., Lopez-Soriano, J., Almendro, V., Busquets, S. & Lopez-Soriano, F. J. Cross-talk between skeletal muscle and adipose tissue: a link with obesity? *Med. Res. Rev.* **25**, 49–65 (2005). 15389734 10.1002/med.20010

[44] Corrales, P., Vidal-Puig, A. & Medina-Gomez, G. PPARs and metabolic disorders associated with challenged adipose tissue plasticity. *Int. J. Mol. Sci.* **19**, 2124 (2018). 10.3390/ijms19072124 PMC6073677 30037087

[45] Ma, X., Wang, D., Zhao, W. & Xu, L. Deciphering the roles of PPARγ in adipocytes via dynamic change of transcription complex. *Front. Endocrinol.* **9**, 473 (2018). 10.3389/fendo.2018.00473 PMC6110914 30186237

[46] He, W. et al. Adipose-specific peroxisome proliferator-activated receptor gamma knockout causes insulin resistance in fat and liver but not in muscle. *Proc. Natl Acad. Sci. USA* **100**, 15712–15717 (2003). 14660788 10.1073/pnas.2536828100 PMC307633

[47] Wang, Z. W. et al. The role of leptin resistance in the lipid abnormalities of aging. *FASEB J.* **15**, 108–114 (2001). 11149898 10.1096/fj.00-0310com

[48] Rocchi, S. et al. A unique PPARγ ligand with potent insulin-sensitizing yet weak adipogenic activity. *Mol. Cell* **8**, 737–747 (2001). 11684010 10.1016/s1097-2765(01)00353-7

[49] Berger, J. P. et al. Distinct properties and advantages of a novel peroxisome proliferator-activated protein [gamma] selective modulator. *Mol. Endocrinol.* **17**, 662–676 (2003). 12554792 10.1210/me.2002-0217

[50] Tontonoz, P. & Spiegelman, B. M. Fat and beyond: the diverse biology of PPARγ. *Annu. Rev. Biochem* **77**, 289–312 (2008). 18518822 10.1146/annurev.biochem.77.061307.091829

[51] Mauvais-Jarvis, F. Sex differences in energy metabolism: natural selection, mechanisms and consequences. *Nat. Rev. Nephrol.* **20**, 56–69 (2024). 37923858 10.1038/s41581-023-00781-2

[52] Saavedra-Pena, R. D. M., Taylor, N., Flannery, C. & Rodeheffer, M. S. Estradiol cycling drives female obesogenic adipocyte hyperplasia. *Cell Rep.* **42**, 112390 (2023). 37053070 10.1016/j.celrep.2023.112390 PMC10567995

[53] Nature journals raise the bar on sex and gender reporting in research. *Nature* **605**, 396 (2022). 10.1038/d41586-022-01218-9 35585338

[54] Zucker, I. & Prendergast, B. J. Sex differences in pharmacokinetics predict adverse drug reactions in women. *Biol. Sex. Differ.* **11**, 32 (2020). 32503637 10.1186/s13293-020-00308-5 PMC7275616

