# Author Correction: Ret finger protein deficiency attenuates adipogenesis in male mice with high fat diet-induced obesity

**DOI:** 10.1038/s12276-025-01568-0

**Published:** 2025-10-06

**Authors:** Yun-Gyeong Lee, Anna Jeong, Yongwoon Lim, Sera Shin, Hosouk Joung, Hye Jung Cho, Su-Jin Lee, Hwang Chan Yu, Hyung-Seok Kim, Kwang-Il Nam, Gwang Hyeon Eom, Byung-Hyun Park, So-Young Park, Duk-Hwa Kwon, Hyun Kook

**Affiliations:** 1 Department of Pharmacology, Chonnam National University Medical School, Hwasun, Republic of Korea; 2 Chonnam University Resaserch Institute of Medical Science, Chonnam National University Medical School, Hwasun, Republic of Korea; 3 BioMedical Sciences Graduate Program, Chonnam National University, Hwasun, Republic of Korea; 4 BK21 plus Center for Creative Biomedical Scientists, Chonnam National University, Gwangju, Republic of Korea; 5 Department of Anatomy, Chonnam National University Medical School, Hwasun, Republic of Korea; 6 Department of Forensic Medicine, Chonnam National University Medical School, Hwasun, Republic of Korea; 7 Graduate School of Medical Science and Engineering, Korea Advanced Institute of Science and Technology, Daejeon, Republic of Korea; 8 Department of Physiology, College of Medicine, Yeungnam University, Daegu, Republic of Korea

**Keywords:** Transcriptional regulatory elements, Obesity, Disease model

Correction to: *Experimental & Molecular Medicine* 10.1038/s12276-025-01553-7, published online 18 September 2025

After online publication of this article, the authors noticed an error in the Fig. 2 section.

In Fig. 2 of the article, the image in the upper-right panel of Fig. 2e (eWAT histology) was inadvertently duplicated as the upper-right panel of Fig. 2f (iWAT histology).

The correct image for Fig. 2f has now been provided. The correction does not affect the interpretation of the in vivo experiments or the overall conclusions of the paper. The original article has been corrected.

Original Fig. 2.
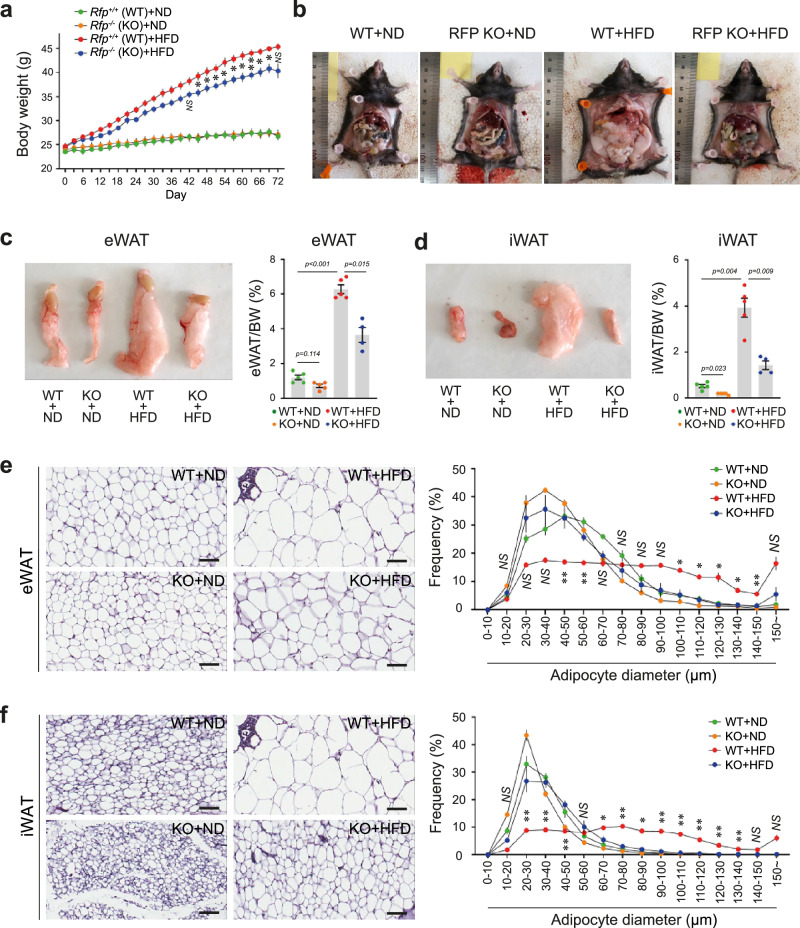


Corrected Fig. 2.
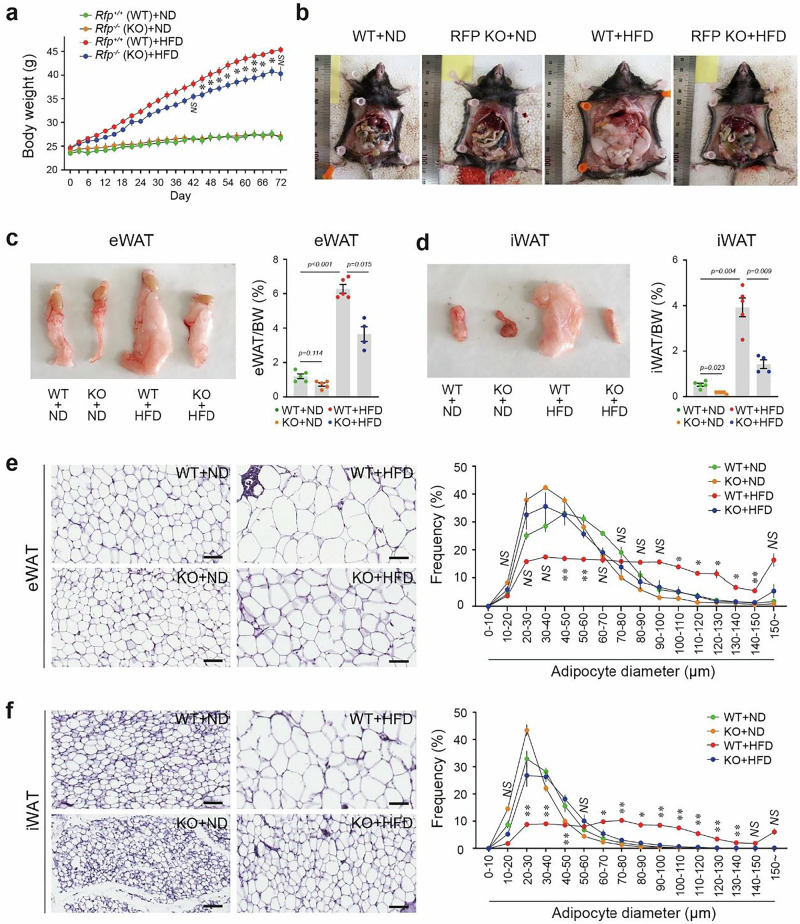


The authors apologize for any inconvenience caused.

