# Supplementary material for: Ret finger protein deficiency attenuates adipogenesis in male mice with high fat diet-induced obesity
Source: Exp Mol Med. 2025 Sep 18;57(9):2052–66. doi: 10.1038/s12276-025-01553-7 (PMC12508092; doi:10.1038/s12276-025-01553-7)
Supplement: Supplementary file 1 — Supplementary Information [file 12276_2025_1553_MOESM1_ESM.pdf]

**Supplementary Information (SI)**

Ret finger protein deficiency attenuates adipogenesis in male mice with high fat diet-induced obesity

Yun-Gyeong Lee[1,2,3,4], Anna Jeong[1,2,4], Yongwoon Lim[1], Sera Shin[1], Hosouk Joung[2], Hye Jung Cho[2,5], Su-Jin Lee[6], Hwang Chan Yu[7], Hyung-Seok Kim[6], Kwang-Il Nam[2,5], Gwang Hyeon Eom[1,2,3], Byung-Hyun Park[7], So-Young Park[8], Duk-Hwa Kwon[1,2,4]*, Hyun Kook[1,2,3,4]*

[1]Department of Pharmacology, Chonnam National University Medical School, Hwasun, Jeollanamdo, Republic of Korea.

[2]Chonnam University Resaserch Institute of Medical Science, Chonnam National University Medical School, Hwasun, Jeollanamdo, Republic of Korea.

[3]BioMedical Sciences Graduate Program (BMSGP), Chonnam National University, Hwasun, Jeollanamdo 58128, Republic of Korea.

[4]BK21 plus Center for Creative Biomedical Scientists, Chonnam National University, Gwangju, Republic of Korea.

[5]Department of Anatomy, Chonnam National University Medical School, Hwasun, Jeollanamdo, Republic of Korea.

[6]Department of Forensic Medicine, Chonnam National University Medical School, Hwasun, Jeollanamdo, Republic of Korea.

[7]Graduate School of Medical Science and Engineering, Korea Advanced Institute of Science and Technology, Daejeon, Republic of Korea.

[8]Department of Physiology, College of Medicine, Yeungnam University, Daegu, Republic of Korea.

* These authors contributed equally to this work

Correspondence to

Dr. Duk-Hwa Kwon, PhD

Department of Pharmacology,

Chonnam National University Medical School

Basic Medical Research Building (M4)

Chonnam National University Biomedical Research Center

264 Seoyang-ro, Hwasun-gun,

Jeollanamdo, 58128,

South Korea

+82-61-379-2841

Email: elio9359@hanmail.net

OR

Prof. Hyun Kook, MD, PhD

Professor

Department of Pharmacology,

Chonnam National University Medical School

Basic Medical Research Building (M4)

Chonnam National University Biomedical Research Center

264 Seoyang-ro, Hwasun-gun,

Jeollanamdo, 58128,

South Korea

+82-61-379-2834

Email: kookhyun@chonnam.ac.kr

**Supplementary Figures**

**Supplementary Table 1. List of primers for siRNA, PCR amplification, and chromatin immunoprecipitation**

List of siRNA sequence

| Name | Sense | Antisense |
|---|---|---|
| *siRFP*-#1 | GAGAGAUGCCAUACCUUUA | UAAAGGUAUGGCAUCUCUC |
| *siRFP*-#2 | GUGGUAUGGGAAAGAAUAU | AUAUUCUUUCCCAUACCAC |

List of primer set sequence

| Name | Forward sequence | Reverse sequence |
|---|---|---|
| *RFP*_human, mouse | CTCAGCCCCCCAGAATGGATTC | CCATGGAATGACCATGATTCCCAA |
| *AP2*_human | CTGGCATGGCCAAACCTAAC | ACGCATTCCACCACCAGTTTA |
| *Adipsin*_human | GACAGCTGCAAGGGTGACTC | CAACCAGATGCAGGAGTGGA |
| *GAPDH*_human | GGACTCATGACCACAGTCCA | TCAGCTCAGGGATGACCTTG |
| *AP2*_mouse | AAAGAAGTGGGAGTGGGCTT | GTGGTCGACTTTCCATCCCA |
| *Adiponectin*_mouse | AAGGACAAGGCCGTTCTCT | TATGGGTAGTTGCAGTCAGTTGG |
| *Plin*_mouse | GGGAAGCATCGAGAAGGTG | TGTCGAGAAAGAGTGTTGGC |
| *Adipsin*_mouse | CCTGAACCCTACAAGCGATG | CAACGAGGCATTCTGGGATAG |
| *Lpl*_mouse | GTAGACTGGTTGTATCGGGC | CTAAGAGGTGGACGTTGTCTAG |
| *GAPDH*_mouse | AACGGATTTGGCCGTATTGG | CATTCTCGGCCTTGACTGTG |

List of primer set sequence for chromatin immunoprecipitation

| Name | Forward sequence | Reverse sequence |
|---|---|---|
| *AP2*_ChIP | ATGTCACAGGCATCTTATCCACC | AACCCTGCCAAAGAGACAGAGG |
| *Adiponectin*_ChIP | GGTGCTGGGAATTGAACTCA | CCTGTTTCCAGGCTTTGGCC |

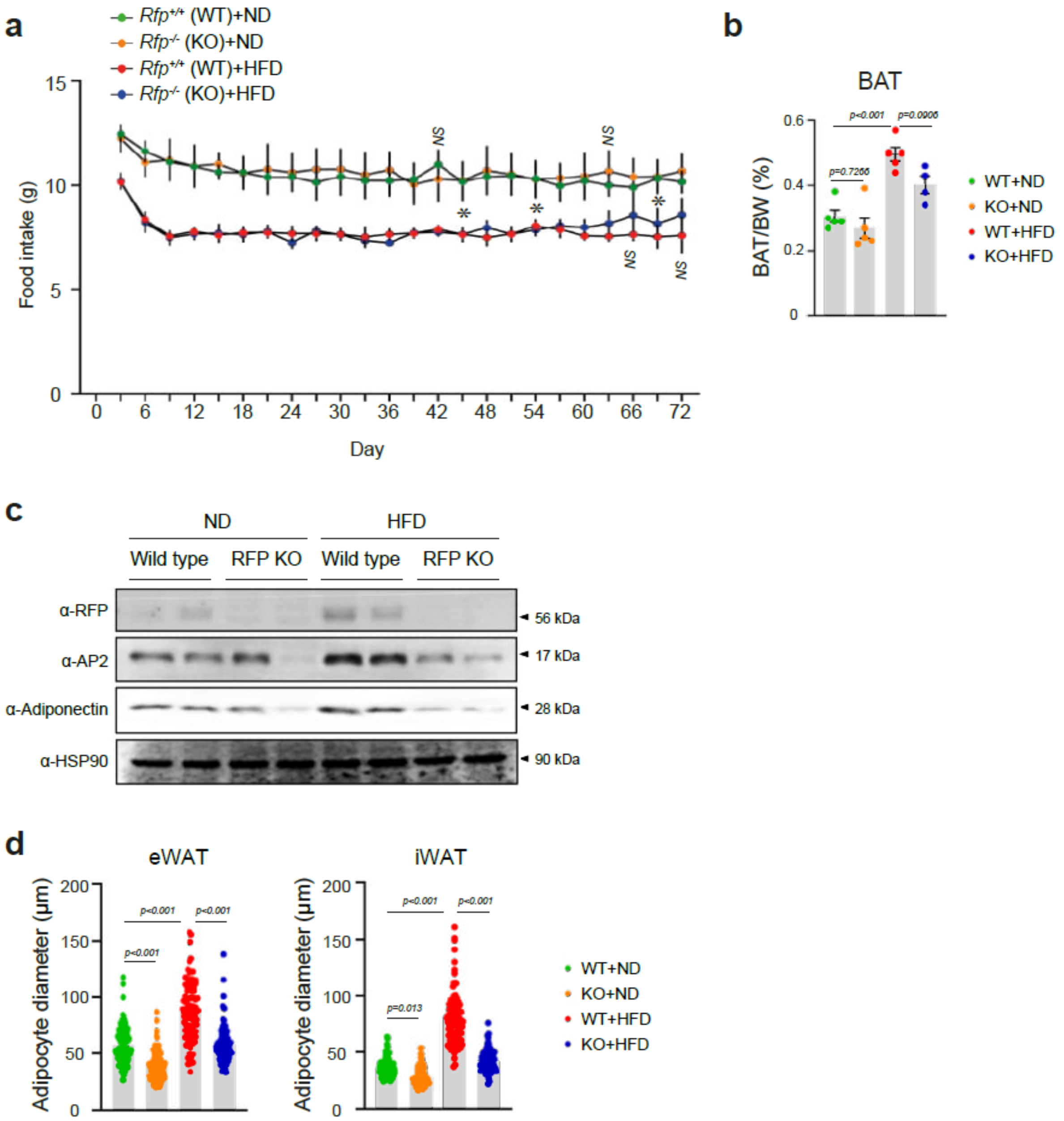


**Supplementary Fig 1. Metabolic phenotyping and adipose tissue characteristics in HFD-fed RFP KO mice. a** Food intake in ND or HFD fed WT and RFP KO mice. n=5. Statistical analysis was performed using one-way ANOVA with post hoc Tukey's test. **b** BAT weight in ND or HFD fed WT and RFP KO mice. n=4~5. Statistical analysis was performed using one-way ANOVA with post hoc Tukey's test. **c** Western blot analysis of adipose tissue from HFD-fed RFP KO mice. **d** Quantitative analysis of adipocyte size in adipose tissue sections from

HFD-fed mice. Statistical analysis was performed using two-way ANOVA with post hoc Tukey's test. All data are presented as mean ± SEM.

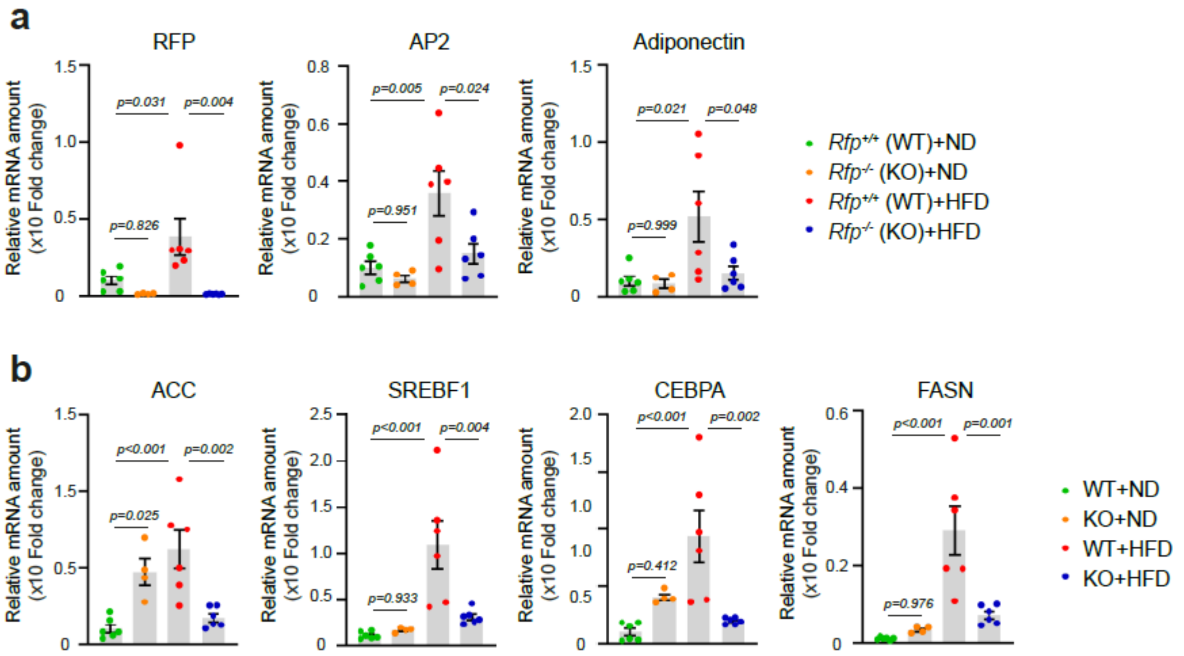


**Supplementary Fig 2. Changes in expression of metabolism-associated genes in HFD-fed RFP KO mice. a** Quantitative PCR (qPCR) analysis of adipogenic genes in adipose tissue from HFD-fed RFP KO mice. n=4~6. Statistical analysis was performed using two-way ANOVA with post hoc Tukey's test. **b** Quantitative PCR (qPCR) analysis of lipogenic genes in adipose tissue from HFD-fed RFP KO mice. n=4~6. All data are presented as mean ± SEM.

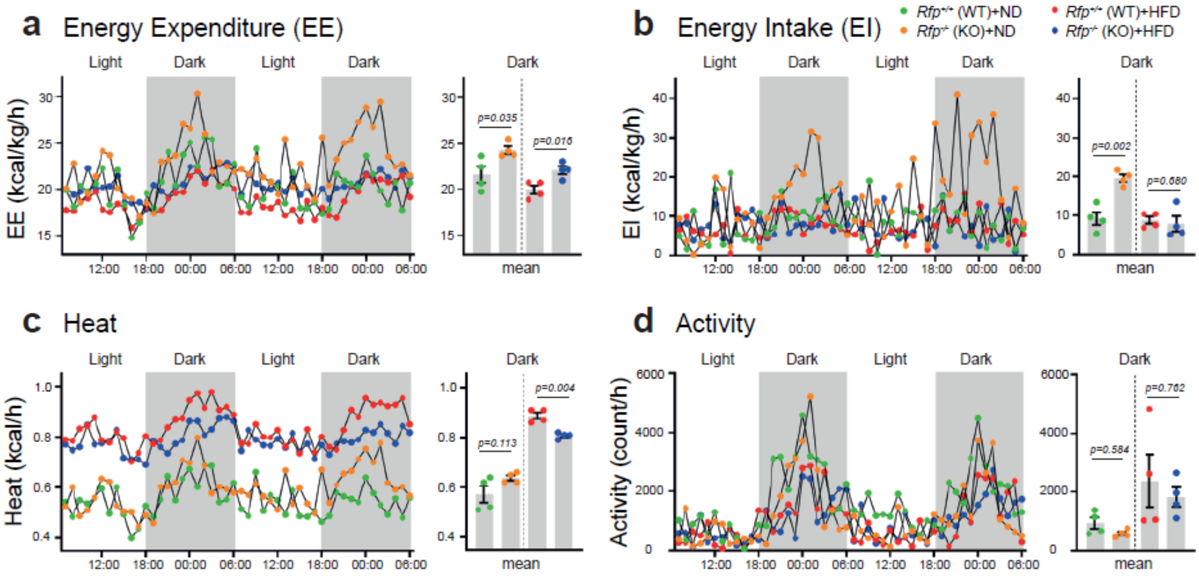


**Supplementary Fig 3. Indirect calorimetry analysis of energy metabolism in HFD-fed RFP KO mice.** Indirect calorimetry data showing energy expenditure (**a**), energy intake (**b**), activity (**c**), and heat production (**d**) in HFD-fed RFP KO mice and WT controls. Statistical analysis was performed using two-way ANOVA with post hoc Tukey's test. All data are presented as mean ± SEM.

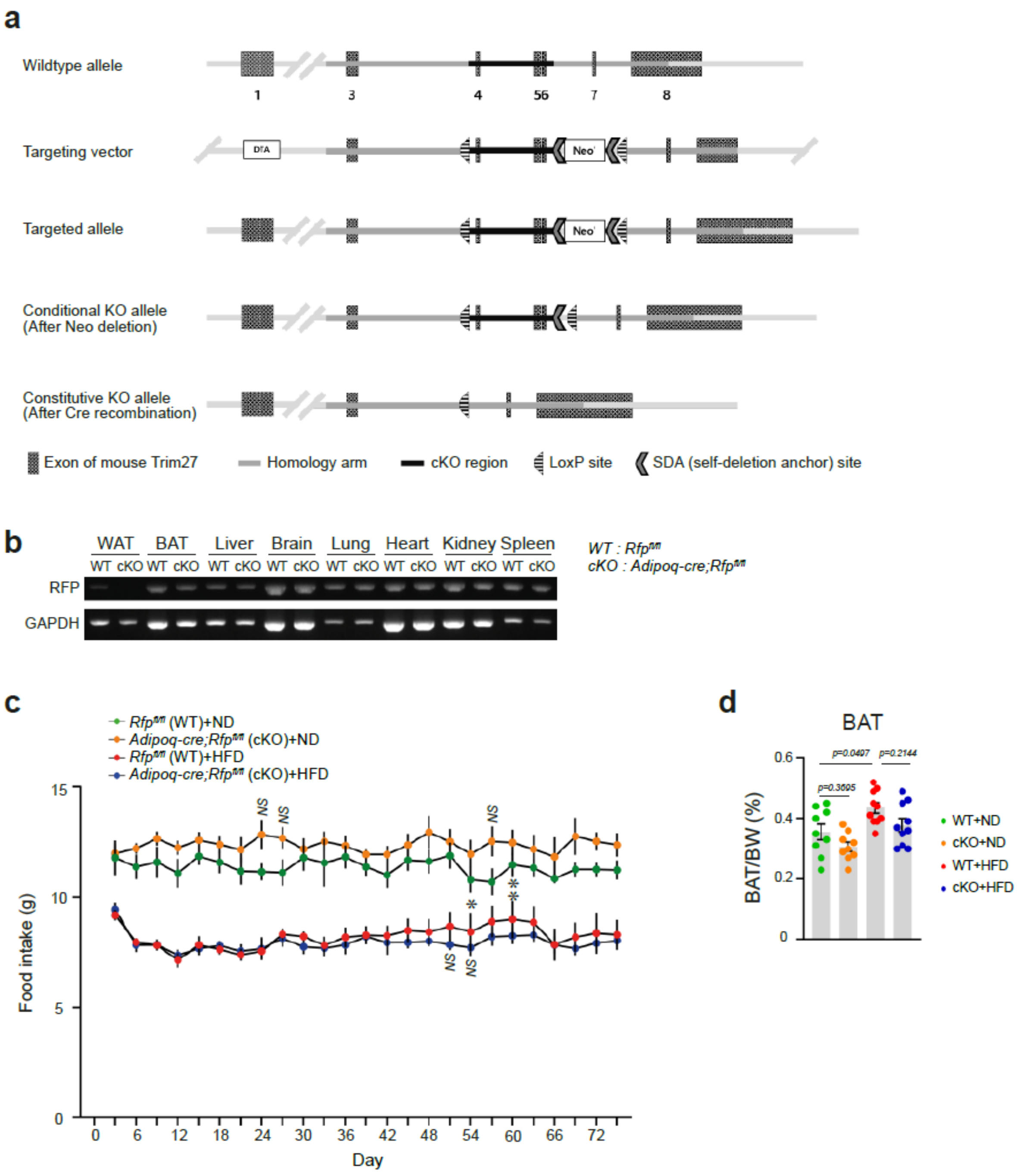


**Supplementary Fig 4. Generation and characterization of adipocyte-specific RFP knockout (RFP cKO) mice. a** RFP cKO mice were generated by crossing *RFP*$^{flox/flox}$ mice with Adipoq-cre mice. **b** Efficiency of RFP deletion in white adipose tissue was confirmed by RT-PCR. **c** Food intake in ND or HFD fed RFP floxed and RFP cKO mice. n=9~10. Statistical analysis was performed using one-way ANOVA with post hoc Tukey's test. **d** BAT

weight in ND or HFD fed RFP floxed and RFP cKO mice. n=9~10. Statistical analysis was performed using one-way ANOVA with post hoc Tukey's test. All data are presented as mean ± SEM.

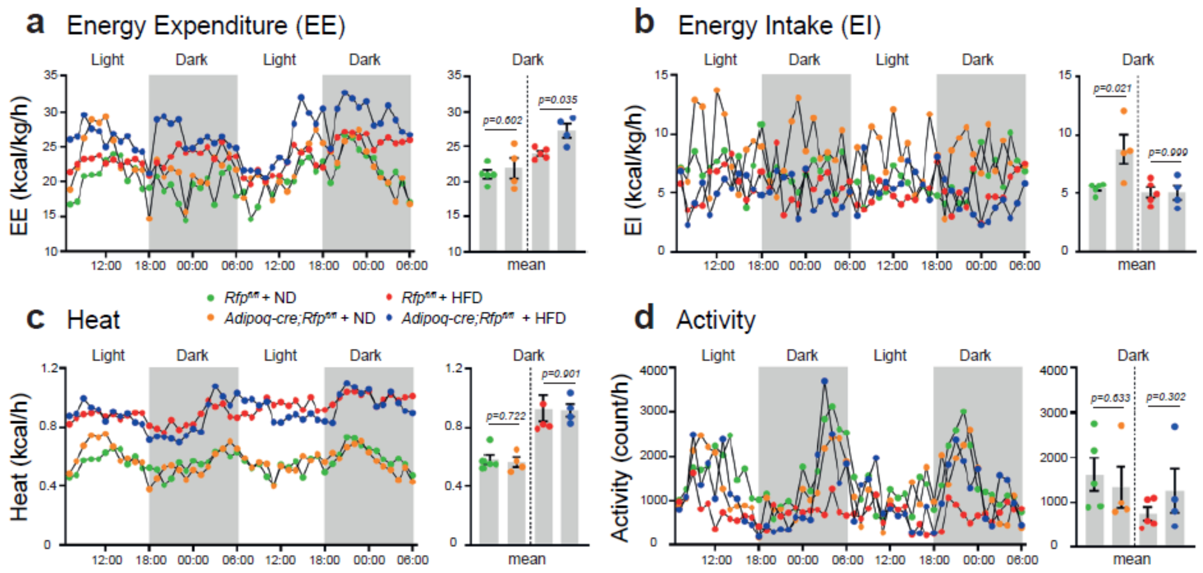


**Supplementary Fig 5. Indirect calorimetry analysis of energy metabolism in HFD-fed adipocyte-specific RFP knockout (RFP cKO) mice.** Indirect calorimetry data showing energy expenditure (**a**), energy intake (**b**), activity (**c**), and heat production (**d**) in HFD-fed RFP cKO mice and RFP floxed mice. n=4~5. Statistical analysis was performed using two-way ANOVA with post hoc Tukey's test. All data are presented as mean ± SEM.

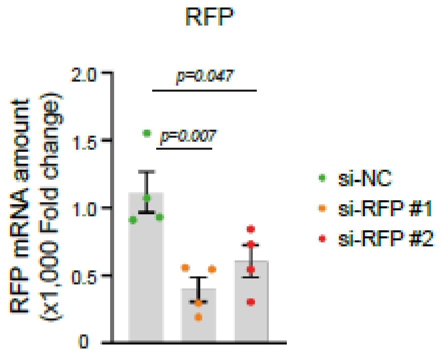


**Supplementary Fig 6. Validation of RFP knockdown in 3T3-L1 cells.** Quantitative PCR (qPCR) analysis confirmed efficient knockdown of RFP in both siRNA-treated groups compared with control group. n=4. Statistical analysis was performed using two-way ANOVA with post hoc Sidak's test. All data are presented as mean ± SEM.

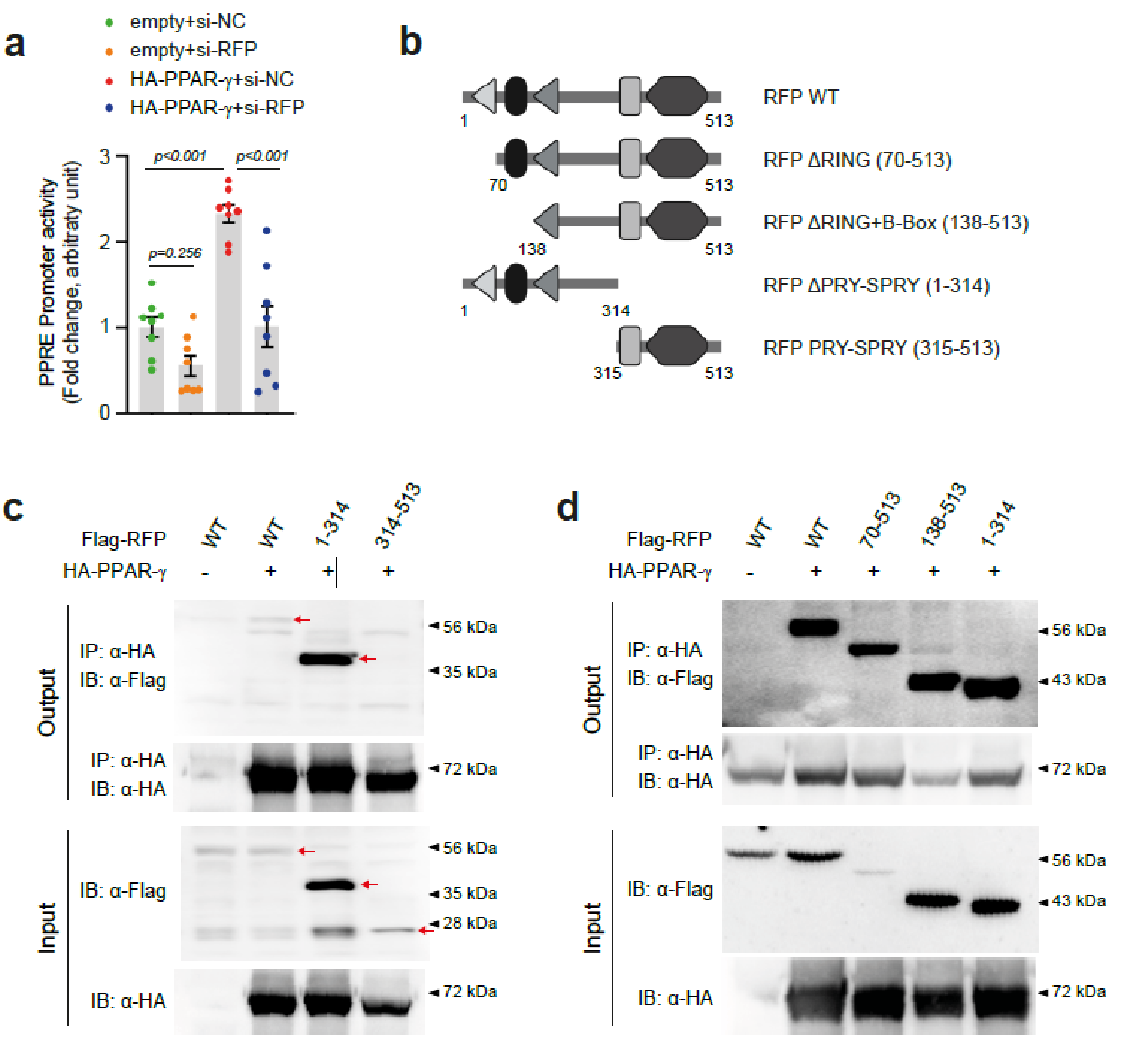


**Supplementary Fig 7. RFP interaction with PPAR-γ and identification of binding domain. a** Effect of RFP knockdown on PPRE promoter activity with direct repeat-1 (DR-1) repeats in 3T3-L1 cells. n=8. Statistical analysis was performed using one-way ANOVA with post hoc Sidak's test. **b** Full-length RFP and deletion constructs (ΔRING: amino acids 70-513; ΔRING+B-Box: amino acids 138-513; ΔPRY-SPRY: amino acids 1-314; PRY-SPRY: amino acids 315-513) were used for domain mapping. **c, d** The coiled-coil domain of RFP plays a critical role in mediating its interaction with PPAR-γ. The red arrow point to the detected band. All data are presented as mean ± SEM.
